# An informatics search for the low-molecular weight chromium-binding peptide

**DOI:** 10.1186/1472-6769-4-2

**Published:** 2004-12-16

**Authors:** Deendayal Dinakarpandian, Vincent Morrissette, Shveta Chaudhary, Kambiz Amini, Brian Bennett, J David Van Horn

**Affiliations:** 1 Division of Computer Science and Electrical Engineering, School of Computing and Engineering, University of Missouri-Kansas City, Kansas City, MO 64110, USA; 2 Department of Chemistry, University of Missouri-Kansas City, 5110 Rockhill Road, Kansas City, MO 64110, USA; 3 National Biomedical EPR Center, Medical College of Wisconsin, Milwaukee, WI 53226 USA

## Abstract

**Background:**

The amino acid composition of a low molecular weight chromium binding peptide (LMWCr), isolated from bovine liver, is reportedly E:G:C:D::4:2:2:2, though its sequence has not been discovered. There is some controversy surrounding the exact biochemical forms and the action of Cr(III) in biological systems; the topic has been the subject of many experimental reports and continues to be investigated. Clarification of Cr-protein interactions will further understanding Cr(III) biochemistry and provide a basis for novel therapies based on metallocomplexes or small molecules.

**Results:**

A genomic search of the non-redundant database for all possible decapeptides of the reported composition yields three exact matches, EDGEECDCGE, DGEECDCGEE and CEGGCEEDDE. The first two sequences are found in ADAM 19 (A Disintegrin and Metalloproteinase domain 19) proteins in man and mouse; the last is found in a protein kinase in rice (*Oryza sativa*). A broader search for pentameric sequences (and assuming a disulfide dimer) corresponding to the stoichiometric ratio E:D:G:C::2:1:1:1, within the set of human proteins and the set of proteins in, or related to, the insulin signaling pathway, yields a match at an acidic region in the α-subunit of the insulin receptor (-EECGD-, residues 175–184). A synthetic peptide derived from this sequence binds chromium(III) and forms a metal-peptide complex that has properties matching those reported for isolated LMWCr and Cr(III)-containing peptide fractions.

**Conclusion:**

The search for an acidic decameric sequence indicates that LMWCr may not be a contiguous sequence. The identification of a distinct pentameric sequence in a significant insulin-signaling pathway protein suggests a possible identity for the LMWCr peptide. This identification clarifies directions for further investigation of LMWCr peptide fractions, chromium bio-coordination chemistry and a possible role in the insulin signaling pathway. Implications for models of chromium action in the insulin-signaling pathway are discussed.

## Background

**Figure 1:**
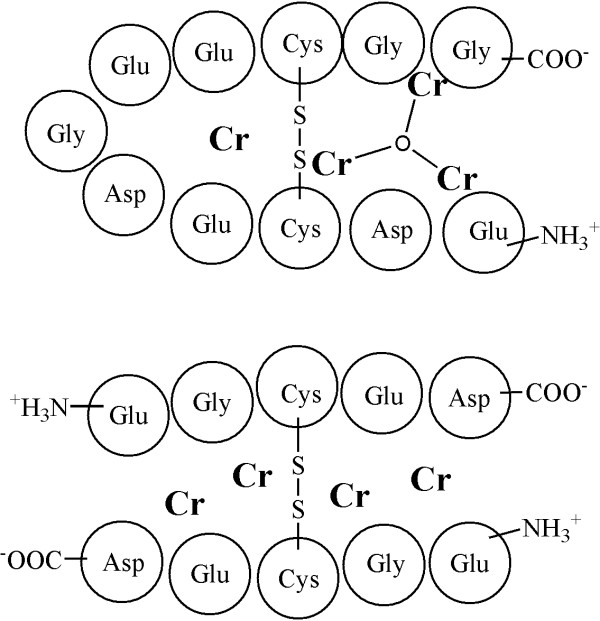
Hypothetical models of low-molecular weight Cr peptide complexes.

Type II diabetes continues to grow as a worldwide epidemic; it is expected that this disease will surpass 300 million cases by 2025 [1-5]. Understanding the complex signaling mechanisms of insulin receptor and downstream factors, e.g. IRS1 and IRS2 [6], is crucial in the design of effective therapeutic strategies. Clarification of such signaling mechanisms is expected to lead to discoveries to cure or prevent diabetes and other metabolic conditions [1]. Current strategies include the search for small synthetic or naturally-derived molecules to act upstream of IRS1, to adequately compensate for insulin dysregulation [7,8]. One unknown facet in this complex problem is the role that chromium may play in the regulation of glucose metabolism; the molecular basis of chromium action in biological systems has not been definitively explained [9,10]. While the toxicology Cr(VI) has been well characterized [11], an understanding of the biochemistry and action of Cr(III) continues to elude researchers within this field [9,11-18]. The lack of good mechanistic models and experiments currently limits researchers' ability to assess the relative importance of Cr(III) and its possible roles in glucose metabolism, obesity or Type II diabetes. Recently, a Cr-peptide fraction isolated from bovine liver [19-23] was shown to potentiate the action of insulin. The amino acid composition of this "low molecular weight Cr" complex (LMWCr, containing 3–4 chromic atoms) was identified to be approximately E:G:C:D::4:2:2:2 [18,21,24], though the sequence remains unknown (Figure 1) and there appears to be some difficulty in exactly repeating this work. Although not specifically related to Cr(III) metabolism, Ramasami and co-workers report that Cr(III) induces structural changes and long range ordering in collagenous tissues and suggest the formation of multinuclear Cr-oxo and hydroxo clusters (2, 3, or 4 Cr atoms) holding the proteinaceous molecular assembly together [25,26]. In this study, we report on a bioinformatics search for the LMWCr peptide, and propose that the peptide that binds to Cr(III) may be the pentameric sequence (-EECGD-) as it is found within the insulin receptor (INSR; Swiss-Prot: P06213) (-NKDDNEECGD-, residues 175–184) and conforms to the predicted stoichiometry. Further, visible absorption data and electron paramagnetic measurements of the synthesized disulfide linked dimeric Cr-peptide complex are identical to that of the biologically isolated fractions.

## Results

### Genomic search for LMWCr

Recent reports [18,21,24], using extracts from bovine liver, suggest that LMWCr is of peptide origin, having an approximate stoichiometric ratio consisting of E:D:G:C::4:2:2:2. In the absence of the exact sequence of this peptide, we generated all possible permutations matching the reported stoichiometry of LMWCr (10!/4!2!2!2! = 18,900) and performed an exact match search of the non-redundant (*nr*) protein database for their occurrence. Based on a simple model where all amino acids are equally possible and about 1.5 million sequences of average length 200 present in the *nr *database, the E-value for an independent exact match is about 0.55 [(0.05)^10 ^* 200 * 1.5 * 10^6 ^* 18,900].

We found perfect matches in the *nr *database corresponding to 3 unique permutations: EDGEECDCGE, DGEECDCGEE and CEGGCEEDDE. One match, CEGGCEEDDE, was found only in one sequence annotated as a putative protein kinase in rice (*Oryza sativa*). The peptide sequences, EDGEECDCGE and DGEECDCGEE, both matched the same region of the disintegrin domain of the human and mouse versions of ADAM 19 (A Disintegrin and Metalloproteinase domain 19; GeneID: 8728) [27]. Since the bovine form of ADAM 19 has yet to be sequenced, we were unable to determine if this sequence is also present in *bos taurus*. This acid-rich sequence region (Table 1) is present at the beginning of the disintegrin domain of ADAM19 [27]. We note that the DNA coding location for this subsequence is at the very end of an exon, giving rise to the speculation that the decameric peptide could possibly be an alternative splicing product. Arguments against this sequence giving rise to LMWCr and the complex acting intracellulary are that ADAM 19 is a membrane protein with extracellular domains. This argument is not needed if Cr might act extracellularly; the conserved nature of the motif leads one to suspect that it plays an important role, but it is at best a highly tentative candidate for being LMWCr. From these results, LMWCr appears unlikely to be a contiguous peptide sequence, unless an as-yet undiscovered peptide is found.

**Table 1:** Multiple sequence alignment showing conservation of the EDGEECDCGE motif in all known mammalian forms of ADAM19 and a homologous putative ADAM in fission yeast (top). Multiple sequence alignment showing proposed Cr(III) binding sequence, NKDDNEECGD, conserved in the insulin receptor (INSR) across species, but not in the insulin-like growth factor receptor (IG1R) (bottom).

SwissProt	Protein	Species	Sequence
Q9H013	ADAM19	Human	GNGYLEDGEECDCGEEEECNNP
O35674	ADAM19	Mouse	GNGYLEDGEECDCGEEEECNNP
	ADAM19	Rat	GNGYLEDGEECDCGEEDECKNP
O13766	Homolog	*S. Pombe*	GNGIVEDGEECDCGEDCENNPC

P15208	INSR	Mouse	NKDDN**EECGD**VCPGT
P15127	INSR	Rat	NKDDN**EECGD**VCPGT
P06213	INSR	Human	NKDDN**EECGD**ICPGT
Q9PVZ4	Homolog	*Xenopus*	NRDNK**EECGD**VCPGT
Q60751	IG1R	Mouse	NKPPKECGDLCPGTL
P24062	IG1R	Rat	NKPPKECGDLCPGTL
P08069	IG1R	Human	NKPPKECGDLCPGTL
O73798	Homolog	*Xenopus*	NKPPKECVDLCPGA.

Noting that the reported stoichiometry consists of even numbers of amino acids, we further considered the possibility that LMWCr might be a disulfide-linked dimer, with each monomeric unit having the composition, E:D:G:C::2:1:1:1. Such a hypothesis is consistent with the limited resolution of the experimental data used to derive the stoichiometry [24] and yields 60 sequence permutations. The expected number of random matches in the *nr *database is much higher, ~10^4^. Restricting this search to the human proteome gave rise to 439 (expected: ~10^2^) matches, with no reason to prefer one over the other.

### Insulin signaling pathway mapping

Considering that LMWCr might be a subsequence of a protein related to "insulin" or known to be involved in the insulin signalling pathway, we compiled a set of 96 such sequences. This set comprised two components: 1) proteins playing a role in the insulin signaling pathway (23 protein sequences were selected) derived from pathway charts, and 2) the set of all protein sequences derived from SwissProt for the search, "insulin + human" (78 entries). The two components were compared for redundancy and the duplicates removed (5 instances).

A cross comparison of the resulting 439 entries using the BLAST query method versus the set of 96 pathway and insulin-related proteins results in a unique match for one of the 60 pentapeptides, EECGD, within the insulin receptor (INSR), residues 180–184 (expected matches: ~10^-1^). Comparison of the pentapeptide set to a more detailed insulin signaling pathway construct [28] yielded the same result.

This sub-sequence lies in the extracellular α-subunit of INSR in an acid-rich region (-NKDDNEECGD-) towards the end of the L1 domain, and at the start of the "cysteine-rich region". A BLAST query [29] for all INSR homologs in the *nr *database shows that this sub-sequence and acidic region is conserved in mouse and rat. Given the location of this acidic sequence within a molecule central to glucose homeostasis, the correspondence with experimentally measured stoichiometry and conservation across multiple species, we speculate that this sequence, or a fragment from this acid rich region may give rise to Cr-peptide fractions isolated from tissue. This suggestion implies that such fractions may not be homogeneous, discrete Cr(III) complexes, i.e. proteolysis may lead to a group of similar peptides that differ by one or more amino acid residues on either side of the Cr binding site. This interpretation differs significantly from reports on the isolation of LMWCr, which attempt to avoid a proteolytic product.

A crystal or solution structure of this region of the insulin receptor has not been determined. However, the crystal structure of a homologous molecule, the insulin-like growth factor receptor (IGR1, GeneID: 124240) has been published [30,31]. Sequence alignment shows that this molecule exhibits a conserved difference in this region, having the subsequence -KECGD- in the same region instead of -EECGD- as found in INSR. The decameric "acidic region" found in INSR is not present in this growth factor receptor – cf. -NKPPKECGDLCPGTL-. The cysteine in -KECGD- forms a disulfide bond with another cysteine residue 28 positions away in the crystal structure. However, we do not know if this is due to the crystallization conditions or representative of the natural form. Further, the insulin receptor possesses 3 additional cysteine residues compared to the insulin-like growth factor receptor, thus the pattern of pairing of cysteine residues to form disulfide bonds may be different in the two receptor types. The observed difference may be great enough to preclude Cr(III) acting on the insulin-like growth factor receptor.

### Chromium-peptide complex synthesis

**Figure 2:**
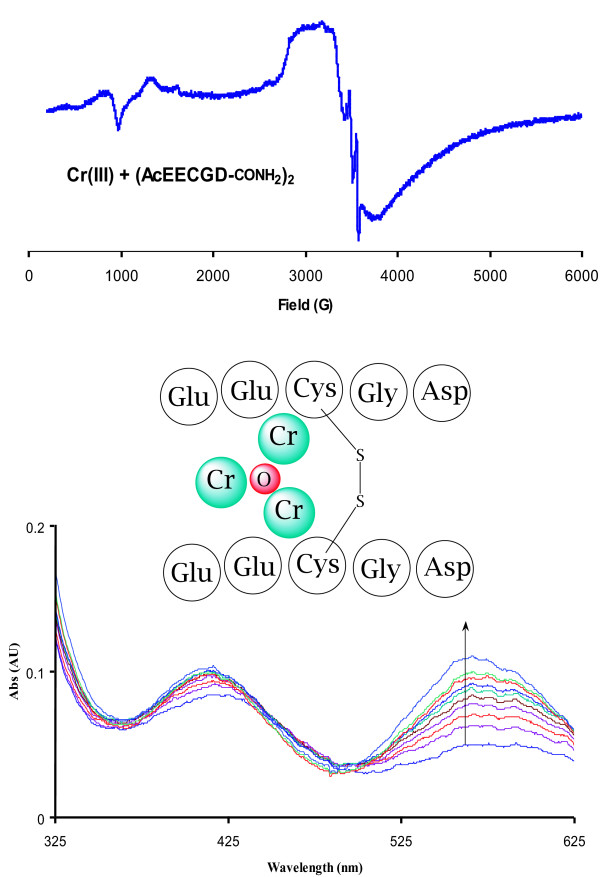
Schematic of proposed Cr-peptide complex. The EPR spectrum (top) is comparable to that of isolated LMWCr with g ~2.0; the spectrum also includes additional hyperfine coupling. Visible absorption spectra of complex formation from the reconstitution of apo-peptide with fresh solutions of chromium chloride (bottom). These data are qualitative and may comprise more than one discreet species.

The identified sequence in hand, we synthesized the pentapeptide (AcEECGD-CONH_2_) and generated the disulfide-linked dimer via air oxidation. This peptide was subjected to conditions for the reconstitution of apo-LMWCr to generate a holo-peptide complex. Incubation of the peptide with fresh solutions of CrCl_3 _results in a clear gray-green solutions of a Cr-peptide complex with visible electronic spectra (Figure 2) consistent with those reported for LMWCr [24]. Further, EPR experiments with Cr-peptide complexes generated from (AcEECGD-CONH_2_)_2 _and (AcNEECGD-CONH_2_)_2 _indicate that Cr exists in trinuclear arrays in these complexes. A preliminary spectrum is shown in Figure 2 consistent with that of isolated LMWCr fractions [32], further characterization is necessary to identify the exact nature of the complexes in these experiments.

## Discussion

Barring the discovery of a novel, unsequenced or unidentified protein or peptide, these data point to the possible sequence of LMWCr fractions and may point to new strategies in therapeutic design. In addition, the question of sequence specificity in Cr(III)-peptide complexes must be fully addressed, along with thermodynamic and kinetic aspects of Cr(III) binding and transfer.

Models of non-toxicological action of Cr(III) in biological systems may broadly fall into three categories: structural, redox, and iron homeostasis. The earliest models [12], and those advanced by Vincent [18], fall into a structural category and focus on the interactions of Cr(III) with peptides and proteins to affect insulin signaling and glucose metabolism, either directly or indirectly. There is an important redox model recently advanced [33] that suggests higher oxidation states of Cr interact with tyrosine phosphatases to inhibit the down-regulation of the insulin receptor. Finally, the chemical similarity of the Cr(III) and Fe(III) cations, and various *in vitro *studies suggest that Cr(III) replacement in the physiological iron transport and storage apparatus may lead to some small beneficial outcome for certain diseases [10]. The biological relevance of these models, and of *in vitro *experiments (including our own) may be finally ascertained only after the fact.

Although unexpected, the results in this report and a critical review of other literature [9,11-18], suggest that an *extracellular *model for Cr(III) biochemistry with respect to insulin signaling may be plausible (see Supporting Information). Such a structural model would include the known aspects of INSR cycling and insulin degradation [34], and include the proposed interactions between Cr(III) and the INSR at the acidic site identified by our genomic search. This model is reminiscent of Mertz and co-workers' original proposal [12] of a ternary interaction between Cr(III), insulin and insulin receptor. It is substantially different from *intracellular *mechanisms for LMWCr action [16,18], redox mechanisms [33], and the iron homeostasis model [10]. In addition, the cycling of the insulin receptor and insulin degradation [34] may satisfy the problems of cellular distribution of Cr(III) and production of LMWCr via proteolysis. Experimentally observed insulin potentiating activity of Cr(III) may result from binding to the alpha subunit or bridging interaction between the two α subunits of an intact INSR molecule.

This model is a parsimonious alternative to current proposals of Cr action in the insulin signaling pathway. However, this model points directly back to significant kinetic and a thermodynamic questions about Cr(III) in biological systems. For instance, what is the physical form of Cr in the bloodstream? How is Cr(III) transported and exchanged between ligands in the serum? Is transport specific? What structure/activity relationship exists in Cr(III) complexes to allow their transport across biological membranes? Thermodynamically, a hydrolyzed, multinuclear Cr cluster should predominate at neutral pH, but transport by transferrin would presumably be in the mononuclear Fe binding sites. Alternatively, Cr(III) clusters may be transported non-specifically in serum by proteins, possibly including transferrin and serum albumin. At this point, there exist significant gaps in understanding the possible biochemistry of Cr(III) and what molecular processes it may affect.

The proposed extracellular model of Cr(III) action in this report is upstream of IRS1, a therapeutic target of White and others [7,8], and may lend itself to small-molecule therapeutic strategies for diabetes and other metabolic conditions [1]. We hope this model may pave the way for innovative experiments, better models of Cr(III) biochemistry and excretion, and further understanding of signaling events in complex biochemical pathways.

## Conclusions

A bioinformatic search for an acidic decameric sequence matching reported stoichiometries of LMWCr amino acid composition indicates that the peptide may not be a contiguous sequence. An expanded search localized a pentameric sequence in the insulin receptor and suggests a possible identity for the Cr(III)-containing peptide fractions derived from liver. Disulfide linked penta- and hexameric peptides based on the identified sequence bind Cr(III) in a similar fashion to LMWCr fractions reported in the literature.

## Methods

### Search for LMWCr

The *nr *database was downloaded and Perl scripts used to search for exact matches corresponding to all unique permutations of EEEEGGCCDD. In a separate search, a set of 78 unique protein sequences from Swiss-Prot were obtained as a result of using the query "insulin human." This set was searched for exact matches corresponding to all unique permutations of EEGCD. In addition, the human proteome was also downloaded and searched for matches to the same set of pentameric peptides.

### N-Acetylglutamylglutamylcysteinylglycylaspartyl carboxamide (AcEECGD-CONH_2_)

The peptide, AcEECGD-CONH_2_, was synthesized by continuous-flow automated solid-phase synthesis on a Perseptive Biosystems Pioneer Peptide Synthesis System. Peptide synthesis was performed by using standard Fmoc-protection strategies with TBTU/DIEA activation strategy. Typically, a 0.5 mmol scale, using Rink amide resin (loading ~0.65 mmol/g) and four times excess of the other reagents (TBTU, protected amino acid) were used. After the solid-phase synthesis was complete, acetylation at the amino terminus was carried out for 2 hours (50:50:1:: acetic anhydride:dimethylformamide:pyridine), and the resulting peptide cleaved from the resin. The peptide was cleaved from the solid phase resin by adding a slurry of 94% trifluoroacetic acid, 2.5% of water, 2.5% of ethanedithiol, and 1% of triisopropylsilane to the reaction vessel and shaking it for 5 h. The trifluoroacetic acid, containing the peptide, was filtered off by vacuum, and the resin was washed 2–3 times with trifluoroacetic acid. The filtrate was evaporated under nitrogen gas until the volume was reduced to 15 mL. 30 mL of ice-cold diethyl ether was added to the filtrate, causing the peptide to precipitate, and the mixture centrifuged to form a pellet of the peptide. The diethyl ether was decanted and the peptide pellet was washed with diethyl ether three times. Finally, the peptide was dissolved in 20 mL water containing 0.1% trifluoroacetic acid and extracted with diethyl ether three times. The aqueous portion was collected and freeze-dried to give the synthetic peptide. AcEECGD-CONH_2_, has a retention time of 5.3 minutes on a 250 mm × 4.5 mm C-18 reversed phase HPLC column using a gradient of 5 to 20% acetonitrile in 0.1% trifluoroacetic acid/water mobile phase running at 1 mL per minute and detected at a wavelength of 220 nm.

### N-Acetylglutamylglutamylcystinylglycylaspartylcarboxamide dimer ((AcEECGD-CONH_2_)_2_)

The synthetic peptide, AcEECGD-CONH_2_, was dissolved to 10 mg/mL in a 0.1 M solution of ammonium carbonate at pH 7 and allowed to oxidize under ambient air for 48 hours. The product was isolated by lyophilization and analyzed by HPLC. The disulfide peptide dimer, (AcEECGD-CONH_2_)_2_, has a retention time of 6.0 minutes using the above conditions. The product, (AcEECGD-CONH_2_)_2_, has a calculated mass of 1183.35141 and exhibits a mass of 1183.3477 Daltons when analyzed by electrospray mass spectrometry.

### Chromium N-acetylglutamylglutamylcystinylglycylaspartylcarboxamide (Cr_3_O(AcEECGD-CONH_2_)_2_)

Fifteen milligrams of (AcEECGD-CONH_2_)_2 _was weighed and dissolved in 30 mL of water. A portion of this solution (5 mL) was taken up in a 25 mL tube, and 4 equivalents of chromium(III) chloride were added as a solid or in an aqueous solution. The reaction of the two components took several minutes and was monitored by ultraviolet-visible spectrophotometry. The chromium peptide complex, Cr_3_O(AcEECGD-CONH_2_)_2_, exhibits characteristic spectral absorbance features at 432 nm and 615 nm. The features are consistent with chromium bound to oxygen atom donors and similar to the reported spectrum of LMWCr [24,32]. EPR spectra were collected using the following parameters: microwave frequency, 9.632 GHz; microwave power incident to the cavity, 2 mW; temperature, 10 K (LHe cryostat). Samples were prepared by incubating a solution of peptide with chromium chloride at a final concentration of 1 mM in metal with excess peptide. The complete characterization (EPR, MS, etc.) of this and analogous Cr-peptide complexes will be reported elsewhere.

## Authors' contributions

DD constructed informatics scripts and searches and made sequence alignments. VM and KA constructed pathway maps and conducted informatics searches. SC assisted in peptide synthesis and conducted HPLC analysis. VM and BB collected ESR spectra. JDVH conceived of the study, synthesized peptides and Cr(III) complexes and drafted the manuscript, tables and graphics. All authors read and approved the final manuscript.

## Supplementary Material

Additional File 1 Supporting figures (3) include HPLC and MS data for AcEECGD-CONH_2 _and proposed Cr(III) model at INSR. Supporting tables (2) include FASTA sequences for insulin signaling map (20 proteins) and pentameric peptides found in genomic search (439 entries) Click here for file
