# Supplementary material for: An informatics search for the low-molecular weight chromium-binding peptide
Source: BMC Chem Biol. 2004 Dec 16;4:2. doi: 10.1186/1472-6769-4-2 (PMC539358; doi:10.1186/1472-6769-4-2)
Supplement: Additional File 1 — Supporting figures (3) include HPLC and MS data for AcEECGD-CONH2 and proposed Cr(III) model at INSR. Supporting tables (2) include FASTA sequences for insulin signaling map (20 proteins) and pentameric peptides found in genomic search (439 entries) [file 1472-6769-4-2-S1.pdf]

# An Informatics Search for the Low-Molecular Weight Chromium-Binding Peptide

Deendayal Dinakarpandian,[1] Vincent Morrissette, Shveta Chaudhary, Kambiz Amini, Brian Bennett,[2] J. David Van Horn*

Department of Chemistry, University of Missouri-Kansas City, 5110 Rockhill Road, Kansas City, MO 64110, USA.

[1] Division of Computer Science and Electrical Engineering, School of Computing and Engineering, University of Missouri-Kansas City, Kansas City, MO 64110, USA.

[3] National Biomedical EPR Center, Medical College of Wisconsin, Milwaukee, WI 53226 USA.

*Corresponding author

Email addresses:

DD: dinakard@umkc.edu

VM: morrissettev@umkc.edu

SC: chaudharys@umkc.edu

KA: kaz54@umkc.edu

BB: bbennett@mcw.edu

JDVH: vanhornd@umkc.edu

## Supporting Figures

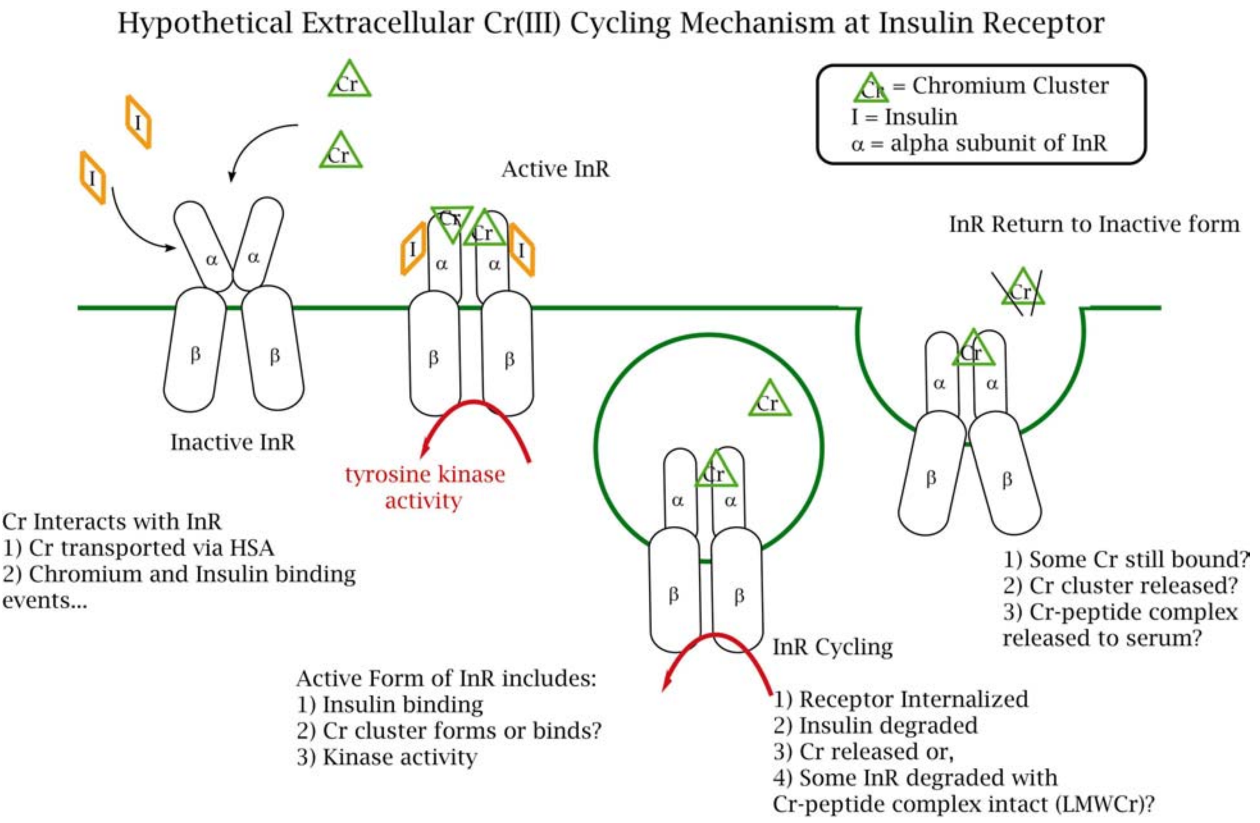


**Figure S1**
Hypothetical Extracellular Cr(III) Cycling Mechanism at Insulin Receptor. Details of Cr(III) serum transport and interactions with serum proteins is not specified in this Figure.

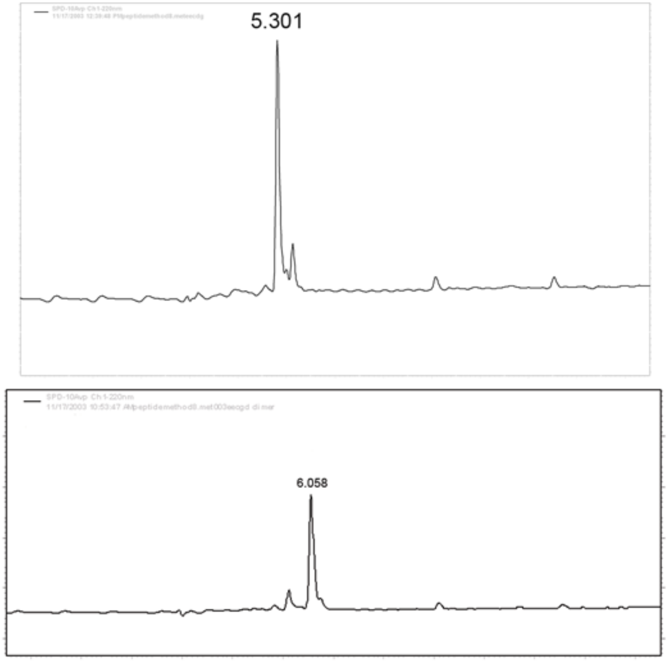


**Figure S2**. HPLC traces of Ac-EECGD-$CONH_2$ (top) and disulfide dimer. See experimental section for conditions.

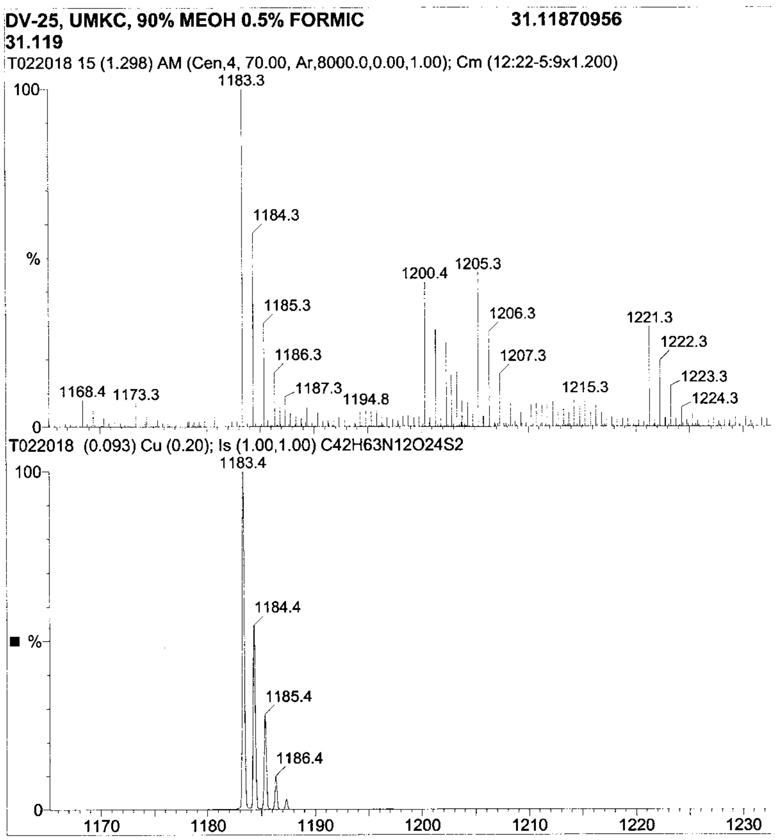


**Figure S3**
ESI-MS spectrum of (AcEECGD-CONH$_2$)$_2$.

## Supporting Materials and Methods:

**Comparison to other sequences related to "insulin".** All protein sequences derived from SwissProt for the search, "insulin human", were compiled and compared for sequence analogy (83 entries) and for duplicates in **Table S2** (5 duplicates). Of the remaining 78 entries, only IGR1 (see text), has sequence similarity to EECGD.

# Supporting Tables

**Table S1**.
Sequence information used in this study for proteins related to the insulin signaling pathway (20 entries). Sequences related to "insulin human" (78 entries), derived from SwissProt, are not shown.

| | |
|---|---|
| 1 | >sp\|P01308\|INS_HUMAN Insulin precursor - Homo sapiens (Human).<br>MALWMRLLPLLALLALWGPDPAAAFVNQHLCGSHLVEALYLVCGERGFFYTPKTRREAED<br>LQVGQVELGGGPGAGSLQPLALEGSLQKRGIVEQCCTSICSLYQLENYCN |
| 2 | >sp\|P06213\|INSR_HUMAN Insulin receptor precursor (EC 2.7.1.112) (IR) (CD220 antigen) - Homo sapiens (Human).<br>MGTGGRRGAAAAPLLVAVAALLLGAAGHLYPGEVCPGMDIRNNLTRLHELENCSVIEGHL<br>QILLMFKTRPEDFRDLSFPKLIMITDYLLLFRVYGLESLKDLFPNLTVIRGSRLFFNYAL<br>VIFEMVHLKELGLYNLMNITRGSVRIEKNNELCYLATIDWSRILDSVEDNYIVLNKDDNE<br>ECGDICPGTAKGKTNCPATVINGQFVERCWTHSHCQKVCPTICKSHGCTAEGLCCHSECL<br>GNCSQPDDPTKCVACRNFYLDGRCVETCPPPYYHFQDWRCVNFSFCQDLHHKCKNSRRQG<br>CHQYVIHNNKCIPECPSGYTMNSSNLLCTPCLGPCPKVCHLLEGEKTIDSVTSAQELRGC<br>TVINGSLIINIRGGNNLAAELEANLGLIEEISGYLKIRRSYALVSLSFFRKLRLIRGETL<br>EIGNYSFYALDNQNLRQLWDWSKHNLTTTQGKLFFHYNPKLCLSEIHKMEEVSGTKGRQE<br>RNDIALKTNGDKASCENELLKFSYIRTSFDKILLRWEPYWPPDFRDLLGFMLFYKEAPYQ<br>NVTEFDGQDACGSNSWTVVDIDPPLRSNDPKSQNHPGWLMRGLKPWTQYAIFVKTLVTFS<br>DERRTYGAKSDIIYVQTDATNPSVPLDPISVSNSSSQIILKWKPPSDPNGNITHYLVFWE<br>RQAEDSELFELDYCLKGLKLPSRTWSPPFESEDSQKHNQSEYEDSAGECCSCPKTDSQIL<br>KELEESSFRKTFEDYLHNVVFVPRKTSSGTGAEDPRPSRKRRSLGDVGNVTVAVPTVAAF<br>PNTSSTSVPTSPEEHRPFEKVVNKESLVISGLRHFTGYRIELQACNQDTPEERCSVAAYV<br>SARTMPEAKADDIVGPVTHEIFENNVVHLMWQEPKEPNGLIVLYEVSYRRYGDEELHLCV<br>SRKHFALERGCRLRGLSPGNYSVRIRATSLAGNGSWTEPTYFYVTDYLDVPSNIAKIIIG<br>PLIFVFLFSVVIGSIYLFLRKRQPDGPLGPLYASSNPEYLSASDVFPCSVYVPDEWEVSR<br>EKITLLRELGQGSFGMVYEGNARDIIKGEAETRVAVKTVNESASLRERIEFLNEASVMKG<br>FTCHHVVRLLGVVSKGQPTLVVMELMAHGDLKSYLRSLRPEAENNPGRPPPTLQEMIQMA<br>AEIADGMAYLNAKKFVHRDLAARNCMVAHDFTVKIGDFGMTRDIYETDYYRKGGKGLLPV<br>RWMAPESLKDGVFTTSSDMWSFGVVLWEITSLAEQPYQGLSNEQVLKFVMDGGYLDQPDN<br>CPERVTDLMRMCWQFNPKMRPTFLEIVNLLKDDLHPSFPEVSFFHSEENKAPESEELEME<br>FEDMENVPLDRSSHCQREEAGGRDGGSSLGFKRSYEEHIPYTHMNGGKKNGRILTLPRSN<br>PS |
| 3 | >sp\|P14672\|GTR4_HUMAN Solute carrier family 2, facilitated glucose transporter, member 4 (Glucose transporter type 4, insulin-responsive) - Homo sapiens (Human).<br>MPSGFQQIGSEDGEPPQQRVTGTLVLAVFSAVLGSLQFGYNIGVINAPQKVIEQSYNETW<br>LGRQGPEGPSSIPPGTLTTLWALSVAIFSVGGMISSFLIGIISQWLGRKRAMLVNNVLAV<br>LGGSLMGLANAAASYEMLILGRFLIGAYSGLTSGLVPMYVGEIAPTHLRGALGTLNQLAI<br>VIGILIAQVLGLESLLGTASLWPLLLGLTVLPALLQLVLLPFCPESPRYLYIIQNLEGPA<br>RKSLKRLTGWADVSGVLAELKDEKRKLERERPLSLLQLLGSRTHRQPLIIAVVLQLSQQL<br>SGINAVFYYSTSIFETAGVGQPAYATIGAGVVNTVFTLVSVLLVERAGRRTLHLLGLAGM<br>CGCAILMTVALLLLERVPAMSYVSIVAIFGFVAFFEIGPGPIPWFIVAELFSQGPRPAAM<br>AVAGFSNWTSNFIIGMGFQYVAEAMGPYVFLLFAVLLLGFFIFTFLRVPETRGRTFDQIS<br>AAFHRTPSLLEQEVKPSTELEYLGPDEND |
| 4 | >sp\|P42336\|P11A_HUMAN Phosphatidylinositol-4,5-bisphosphate 3-kinase catalytic subunit, alpha isoform (EC 2.7.1.153) (PI3-kinase p110 subunit alpha) (PtdIns- 3-kinase p110) (PI3K) - Homo sapiens (Human).<br>MPPRPSSGELWGIHLMPPRILVECLLPNGMIVTLECLREATLVTIKHELFKEARKYPLHQ<br>LLQDESSYIFVSVTQEAEREEFFDETRRLCDLRLFQPFLKVIEPVGNREEKILNREIGFA<br>IGMPVCEFDMVKDPEVQDFRRNILNVCKEAVDLRDLNSPHSRAMYVYPPHVESSPELPKH<br>IYNKLDRGQIIVVIWVIVSPNNDKQKYTLKINHDCVPEQVIAEAIRKKTRSMLLSSEQLK<br>LCVLEYQGKYILKVCGCDEYFLEKYPLSQYKYIRSCIMLGRMPNLKMMAKESLYSQLPMD<br>CFTMPSYSRRISTATPYMNGETSTKSLWVINRALRIKILCATYVNLNIRDIDKIYVRTGI<br>YHGGEPLCDNVNTQRVPCSNPRWNEWLNYDIYIPDLPRAARLCLSICSVKGRKGAKEEHC<br>PLAWGNINLFDYTDTLVSGKMALNLWPVPHGLEDLLNPIGVTGSNPNKETPCLELEFDWF |

| | |
|---|---|
| | SSVVKFPDMSVIEEHANWSVSREAGFSYSHAGLSNRLARDNELRENDKEQLKAISTRDPL<br>SEITEQEKDFLWSHRHYCVTIPEILPKLLLSVKWNSRDEVAQMYCLVKDWPPIKPEQAME<br>LLDCNYPDPMVRGFAVRCLEKYLTDDKLSQYLIQLVQVLKYEQYLDNLLVRFLLKKALTN<br>QRIGHFFFWHLKSEMHNKTVSQRFGLLLESYCRACGMYLKHLNRQVEAMEKLINLTDILK<br>QERKDETQKVQMKFLVEQMRRPDFMDALQGLLSPLNPAHQLGNLRLKECRIMSSAKRPLW<br>LNWENPDIMSELLFQNNEIIFKNGDDLRQDMLTLQIIRIMENIWQNQGLDLRMLPYGCLS<br>IGDCVGLIEVVRNSHTIMQIQCKGGLKGALQFNSHTLHQWLKDKNKGEIYDAAIDLFTRS<br>CAGYCVATFILGIGDRHNSNIMVKDDGQLFHIDFGHFLDHKKKKFGYKRERVPFVLTQDF<br>LIVISKGAQECTKTREFERFQEMCYKAYLAIRQHANLFINLFSMMLGSGMPELQSFDDIA<br>YIRKTLALDKTEQEALEYFMKQMNDAHHGGWTTKMDWIFHTIKQHALN |
| 5 | >sp\|Q9Y4H2\|IRS2_HUMAN Insulin receptor substrate-2 (IRS-2) - Homo sapiens (Human).<br>MASPPRHGPPGPASGDGPNLNNNNNNNNNHSVRKCGYLRKQKHGHKRFFVLRGPGAGGDKA<br>TAGGGSAPQPPRLEYYESEKNWRSKAGAPKRVIALDCCLNINKRADPKHKYLIALYTKDE<br>YFAVAAENEQEQEGWYRALTDLVSEGRAAAGDAPPAAAPAASCSASLPGAVGGSAGAAGA<br>EDSYGLVAPATAAYREVWQVNLKPKGLGQSKNLTGVYRLCLSARTIGFVKLNCEQPSVTL<br>QLMNIRRCGHSDSFFFIEVGRSAVTGPGELWMQADDSVVAQNIHETILEAMKALKELFEF<br>RPRSKSQSSGSSATHPISVPGARRHHHLVNLPPSQTGLVRRSRTDSLAATPPAAKCSSCR<br>VRTASEGDGGAAAGAAAAGARPVSVAGSPLSPGPVRAPLSRSHTLIGGCRAAGTKWHCFP<br>AGGGLQHSRSMSMPVEHLPPAATSPGSLSSSSDHGWGSYPPPPGPHPLLPHPLHHGPGQR<br>PSSGSASASGSPSDPGFMSLDEYGSSPGDLRAFCSHRSNTPESIAETPPARDGGGGGEFY<br>GYMTMDRPLSHCGRSYRRVSGDAAQDLDRGLRKRTYSLTTPARQRPVPQPSSASLDEYTL<br>MRATFSGSAGRLCPSCPASSPKVAYHPYPEDYGDIEIGSHRSSSSNLGADDGYMPMTPGA<br>ALAGSGSGSCRSDDYMPMSPASVSAPKQILQPRAAAAAAAAVPFAGPAGPAPTFAAGRTF<br>PASGGGYKASSPAESSPEDSGYMRMWCGSKLSMEHADGKLLPNGDYLNVSPSDAVTTGTP<br>PDFFSAALHPGGEPLRGVPGCCYSSLPRSYKAPYTCGGDSDQYVLMSSPVGRILEEERLE<br>PQATPGPTQAASAFGAGPTQPPHPVVPSPVRPSGGRPEGFLGQRGRAVRPTRLSLEGLPS<br>LPSMHEYPLPPEPKSPGEYINIDFGEPGARLSPPAPPLLASAASSSSLLSASSPALSLGS<br>GTPGTSSDSRQRSPLSDYMNLDFSSPKSPKPGAPSGHPVGSLDGLLSPEASSPYPPLPPR<br>PSASPSSSLQPPPPPPAPGELYRLPPASAVATAQGPGAASSLSSDTGDNGDYTEMAFGVA<br>ATPPQPIAAPPKPEAARVASPTSGVKRLSLMEQVSGVEAFLQASQPPDPHRGAKVIRADP<br>QGGRRRHSSETFSSTTTVTPVSPSFAHNPKRHNSASVENVSLRKSSEGGVGVGPGGGDEP<br>PTSPRQLQPAPPLAPQGRPWTPGQPGGLVGCPGSGGSPMRRETSAGFQNGLKYIAIDVRE<br>EPGLPPQPQPPPPPLPQPGDKSSWGRTRSLGGLISAVGVGSTRGGCGGPGPGAPAPCPTT<br>YAQH |
| 6 | >sp\|P29354\|GRB2_HUMAN Growth factor receptor-bound protein 2 (GRB2 adapter protein) (SH2/SH3 adapter GRB2) (ASH protein) - Homo sapiens (Human), and Rattus norvegicus (Rat).<br>MEAIAKYDFKATADDELSFKRGDILKVLNEECDQNWYKAELNGKDGFIPKNYIEMKPHPW<br>FFGKIPRAKAEEMLSKQRHDGAFLIRESESAPGDFSLSVKFGNDVQHFKVLRDGAGKYFL<br>WVVKFNSLNELVDYHRSTSVSRNQQIFLRDIEQVPQQPTYVQALFDFDPQEDGELGFRRG<br>DFIHVMDNSDPNWWKGACHGQTGMFPRNYVTPVNRNV |
| 7 | >sp\|P29353\|SHC_HUMAN SHC transforming protein - Homo sapiens (Human).<br>MDLLPPKPKYNPLRNESLSSLEEGASGSTPPEELPSPSASSLGPILPPLPGDDSPTTLCS<br>FFPRMSNLRLANPAGGRPGSKGEPGRAADDGEGIDGAAMPESGPLPLLQDMNKLSGGGGR<br>RTRVEGGQLGGEEWTRHGSFVNKPTRGWLHPNDKVMGPGVSYLVRYMGCVEVLQSMRALD<br>FNTRTQVTREAISLVCEAVPGAKGATRRRKPCSRPLSSILGRSNLKFAGMPITLTVSTSS<br>LNLMAADCKQIIANHHMQSISFASGGDPDTAEYVAYVAKDPVNQRACHILECPEGLAQDV<br>ISTIGQAFELRFKQYLRNPPKLVTPHDRMAGFDGSAWDEEEEEPPDHQYYNDFPGKEPPL<br>GGVVDMRLREGAAPGAARPTAPNAQTPSHLGATLPVGQPVGGDPEVRKQMPPPPPCPGRE<br>LFDDPSYVNVQNLDKARQAVGGAGPPNPAINGSAPRDLFDMKPFEDALRVPPPPQSVSMA<br>EQLRGEPWFHGKLSRREAEALLQLNGDFLVRESTTTPGQYVLTGLQSGQPKHLLLVDPEG<br>VVRTKDHRFESVSHLISYHMDNHLPIISAGSELCLQQPVERKL |
| 8 | >sp\|Q07889\|SOS1_HUMAN Son of sevenless protein homolog 1 (SOS-1) - Homo sapiens (Human).<br>MQAQQLPYEFFSEENAPKWRGLLVPALKKVQGQVHPTLESNDDALQYVEELILQLLNMLC<br>QAQPRSASDVEERVQKSFPHPIDKWAIADAQSAIEKRKRRNPLSLPVEKIHPLLKEVLGY<br>KIDHQVSVYIVAVLEYISADILKLVGNYVRNIRHYEITKQDIKVAMCADKVLMDMFHQDV<br>EDINILSLTDEEPSTSGEQTYYDLVKAFMAEIRQYIRELNLIIKVFREPFVSNSKLFSAN |

| | |
|---|---|
| | DVENIFSRIVDIHELSVKLLGHIEDTVEMTDEGSPHPLVGSCFEDLAEELAFDPYESYAR<br>DILRPGFHDRFLSQLSKPGAALYLQSIGEGFKEAVQYVLPRLLLAPVYHCLHYFELLKQL<br>EEKSEDQEDKECLKQAITALLNVQSGMEKICSKSLAKRRLSESACRFYSQQMKGKQLAIK<br>KMNEIQKNIDGWEGKDIGQCCNEFIMEGTLTRVGAKHERHIFLFDGLMICCKSNHGQPRL<br>PGASNAEYRLKEKFFMRKVQINDKDDTNEYKHAFEIILKDENSVIFSAKSAEEKNNWMAA<br>LISLQYRSTLERMLDVTMLQEEKEEQMRLPSADVYRFAEPDSEENIIFEENMQPKAGIPI<br>IKAGTVIKLIERLTYHMYADPNFVRTFLTTYRSFCKPQELLSLIIERFEIPEPEPTEADR<br>IAIENGDQPLSAELKRFRKEYIQPVQLRVLNVCRHWVEHHFYDFERDAYLLQRMEEFIGT<br>VRGKAMKKWVESITKIIQRKKIARDNGPGHNITFQSSPPTVEWHISRPGHIETFDLLTLH<br>PIEIARQLTLLESDLYRAVQPSELVGSVWTKEDKEINSPNLLKMIRHTTNLTLWFEKCIV<br>ETENLEERVAVVSRIIEILQVFQELNNFNGVLEVVSAMNSSPVYRLDHTFEQIPSRQKKI<br>LEEAHELSEDHYKKYLAKLRSINPPCVPFFGIYLTNILKTEEGNPEVLKRHGKELINFSK<br>RRKVAEITGEIQQYQNQPYCLRVESDIKRFFENLNPMGNSMEKEFTDYLFNKSLEIEPRN<br>PKPLPRFPKKYSYPLKSPGVRPSNPRPGTMRHPTPLQQEPRKISYSRIPESETESTASAP<br>NSPRTPLTPPPASGASSTTDVCSVFDSDHSSPFHSSNDTVFIQVTLPHGPRSASVSSISL<br>TKGTDEVPVPPPVPPRRRPESAPAESSPSKIMSKHLDSPPAIPPRQPTSKAYSPRYSISD<br>RTSISDPPESPPLLPPREPVRTPDVFSSSPLHLQPPPLGKKSDHGNAFFPNSPSPFTPPP<br>PQTPSPHGTRRHLPSPPLTQEVDLHSIAGPPVPPRQSTSQHIPKLPPKTYKREHTHPSMH<br>RDGPPLLENAHSS |
| 9 | >sp\|P01112\|RASH_HUMAN Transforming protein p21/H-Ras-1 (c-H-ras) - Homo sapiens (Human).<br>MTEYKLVVVGAGGVGKSALTIQLIQNHFVDEYDPTIEDSYRKQVVIDGETCLLDILDTAG<br>QEEYSAMRDQYMRTGEGFLCVFAINNTKSFEDIHQYREQIKRVKDSDDVPMVLVGNKCDL<br>AARTVESRQAQDLARSYGIPYIETSAKTRQGVEDAFYTLVREIRQHKLRKLNPPDESGPG<br>CMSCKCVLS |
| 10 | >sp\|P20936\|RSG1_HUMAN Ras GTPase-activating protein 1 (GTPase-activating protein) (GAP) (Ras p21 protein activator) (p120GAP) (RasGAP) - Homo sapiens (Human).<br>MMAAEAGSEEGGPVTAGAGGGGAAAGSSAYPAVCRVKIPAALPVAAAPYPGLVETGVAGT<br>LGGGAALGSEFLGAGSVAGALGGAGLTGGGTAAGVAGAAAGVAGAAVAGPSGDMALTKLP<br>TSLLAETLGPGGGFPPLPPPPYLPPLGAGLGTVDEGDSLDGPEYEEEEVAIPLTAPPTNQ<br>WYHGKLDRTIAEERLRQAGKSGSYLIRESDRRPGSFVLSFLSQMNVVNHFRIIAMCGDYY<br>IGGRRFSSLSDLIGYYSHVSCLLKGEKLLYPVAPPEPVEDRRRVRAILPYTKVPDTDEIS<br>FLKGDMFIVHNELEDGWMWVTNLRTDEQGLIVEDLVEEVGREEDPHEGKIWFHGKISKQE<br>AYNLLMTVGQVCSFLVRPSDNTPGDYSLYFRTNENIQRFKICPTPNNQFMMGGRYYNSIG<br>DIIDHYRKEQIVEGYYLKEPVPMQDQEQVLNDTVDGKEIYNTIRRKTKDAFYKNIVKKGY<br>LLKKGKGKRWKNLYFILEGSDAQLIYFESEKRATKPKGLIDLSVCSVYVVHDSLFGRPNC<br>FQIVVQHFSEEHYIFYFAGETPEQAEDWMKGLQAFCNLRKSSPGTSNKRLRQVSSLVLHI<br>EEAHKLPVKHFTNPYCNIYLNSVQVAKTHAREGQNPVWSEEFVFDDLPPDINRFEITLSN<br>KTKKSKDPDILFMRCQLSRLQKGHATDEWFLLSSHIPLKGIEPGSLRVRARYSMEKIMPE<br>EEYSEFKELILQKELHVVYALSHVCGQDRTLLASILLRIFLHEKLESLLLCTLNDREISM<br>EDEATTLFRATTLASTLMEQYMKATATQFVHHALKDSILKIMESKQSCELSPSKLEKNED<br>VNTNLTHLLNILSELVEKIFMASEILPPTLRYIYGCLQKSVQHKWPTNTTMRTRVVSGFV<br>FLRLICPAILNPRMFNIISDSPSPIAARTLILVAKSVQNLANLVEFGAKEPYMEGVNPFI<br>KSNKHRMIMFLDELGNVPELPDTTEHSRTDLSRDLAALHEICVAHSDELRTLSNERGAQQ<br>HVLKKLLAITELLQQKQNQYTKTNDVR |
| 11 | >sp\|Q9UHA4\|MKI1_HUMAN Mitogen-activated protein kinase kinase 1 interacting protein 1 (MEK binding partner 1) (Mp1) (PRO2783) - Homo sapiens (Human).<br>MADDLKRFLYKKLPSVEGLHAIVVSDRDGVPVIKVANDNAPEHALRPGFLSTFALATDQG<br>SKLGLSKNKSIICYYNTYQVVQFNRLPLVVSFIASSSANTGLIVSLEKELAPLFEELRQV<br>VEVS |
| 12 | >sp\|Q02750\|MPK1_HUMAN Dual specificity mitogen-activated protein kinase kinase 1 (EC 2.7.1.-) (MAP kinase kinase 1) (MAPKK 1) (ERK activator kinase 1) (MAPK/ERK kinase 1) (MEK1) - Homo sapiens (Human).<br>PKKKPTPIQLNPAPDGSAVNGTSSAETNLEALQKKLEELELDEQQRKRLEAFLTQKQKVG<br>ELKDDDFEKISELGAGNGGVVFKVSHKPSGLVMARKLIHLEIKPAIRNQIIRELQVLHEC<br>NSPYIVGFYGAFYSDGEISICMEHMDGGSLDQVLKKAGRIPEQILGKVSIAVIKGLTYLR<br>EKHKIMHRDVKPSNILVNSRGEIKLCDFGVSGQLIDSMANSFVGTRSYMSPERLQGTHYS |

| | |
|---|---|
| | VQSDIWSMGLSLVEMAVGRYPIPPPDAKELELMFGCQVEGDAAETPPRPRTPGRPLSSYG<br>MDSRPPMAIFELLDYIVNEPPPKLPSGVFSLEFQDFVNKCLIKNPAERADLKQLMVHAFI<br>KRSDAEEVDFAGWLCSTIGLNQPSTPTHAAGV |
| 13 | >sp\|P27361\|MK03_HUMAN Mitogen-activated protein kinase 3 (EC 2.7.1.37) (Extracellular signal-regulated kinase 1) (ERK-1) (Insulin-stimulated MAP2 kinase) (MAP kinase 1) (MAPK 1) (p44-ERK1) (ERT2) (p44-MAPK) (Microtubule- associated protein-2 kinase) - Homo sapiens (Human).<br>MAAAAAQGGGGGEPRRTEGVGPGVPGEVEMVKGQPFDVGPRYTQLQYIGEGAYGMVSSAY<br>DHVRKTRVAIKKISPFEHQTYCQRTLREIQILLRFRHENVIGIRDILRASTLEAMRDVYI<br>VQDLMETDLYKLLKSQQLSNDHICYFLYQILRGLKYIHSANVLHRDLKPSNLLINTTCDL<br>KICDFGLARIADPEHDHTGFLTEYVATRWYRAPEIMLNSKGYTKSIDIWSVGCILAEMLS<br>NRPIFPGKHYLDQLNHILGILGSPSQEDLNCIINMKARNYLQSLPSKTKVAWAKLFPKSD<br>SKALDLLDRMLTFNPNKRITVEEALAHPYLEQYYDPTDEPVAEEPFTFAMELDDLPKERL<br>KELIFQETARFQPGVLEAP |
| 14 | >sp\|P19419\|ELK1_HUMAN ETS-domain protein ELK-1 - Homo sapiens (Human).<br>MDPSVTLWQFLLQLLREQGNGHIISWTSRDGGEFKLVDAEEVARLWGLRKNKTNMNYDKL<br>SRALRYYYDKNIIRKVSGQKFVYKFVSYPEVAGCSTEDCPPQPEVSVTSTMPNVAPAAIH<br>AAPGDTVSGKPGTPKGAGMAGPGGLARSSRNEYMRSGLYSTFTIQSLQPQPPPHPRPAVV<br>LPSAAPAGAAAPPSGSRSTSPSPLEACLEAEEAGLPLQVILTPPEAPNLKSEELNVEPGL<br>GRALPPEVKVEGPKEELEVAGERGFVPETTKAEPEVPPQEGVPARLPAVVMDTAGQAGGH<br>AASSPEISQPQKGRKPRDLELPLSPSLLGGPGPERTPGSGSGSGLQAPGPALTPSLLPTH<br>TLTPVLLTPSSLPPSIHFWSTLSPIAPRSPAKLSFQFPSSGSAQVHIPSISVDGLSTPVV<br>LSPGPQKP |
| 15 | >sp\|Q06124\|PTNB_HUMAN Protein-tyrosine phosphatase, non-receptor type 11 (EC 3.1.3.48) (Protein-tyrosine phosphatase 2C) (PTP-2C) (PTP-1D) (SH-PTP3) (SH- PTP2) (SHP-2) - Homo sapiens (Human).<br>MTSRRWFHPNITGVEAENLLLTRGVDGSFLARPSKSNPGDFTLSVRRNGAVTHIKIQNTG<br>DYYDLYGGEKFATLAELVQYYMEHHGQLKEKNGDVIELKYPLNCADPTSERWFHGHLSGK<br>EAEKLLTEKGKHGSFLVRESQSHPGDFVLSVRTGDDKGESNDGKSKVTHVMIRCQELKYD<br>VGGGERFDSLTDLVEHYKKNPMVETLGTVLQLKQPLNTTRINAAEIESRVRELSKLAETT<br>DKVKQGFWEEFETLQQQECKLLYSRKEGQRQENKNKNRYKNILPFDHTRVVLHDGDPNEP<br>VSDYINANIIMPEFETKCNNSKPKKSYIATQGCLQNTVNDFWRMVFQENSRVIVMTTKEV<br>ERGKSKCVKYWPDEYALKEYGVMRVRNVKESAAHDYTLRELKLSKVGQGNTERTVWQYHF<br>RTWPDHGVPSDPGGVLDFLEEVHHKQESIMDAGPVVVHCSAGIGRTGTFIVIDILIDIIR<br>EKGVDCDIDVPKTIQMVRSQRSGMVQTEAQYRFIYMAVQHYIETLQRRIEEEQKSKRKGH<br>EYTNIKYSLADQTSGDQSPLPPCTPTPPCAEMREDSARVYENVGLMQQQKSFR |
| 16 | >sp\|P01100\|FOS_HUMAN Proto-oncogene protein c-fos (Cellular oncogene fos) (G0/G1 switch regulatory protein 7) - Homo sapiens (Human).<br>MMFSGFNADYEASSSRCSSASPAGDSLSYYHSPADSFSSMGSPVNAQDFCTDLAVSSANF<br>IPTVTAISTSPDLQWLVQPALVSSVAPSQTRAPHPFGVPAPSAGAYSRAGVVKTMTGGRA<br>QSIGRRGKVEQLSPEEEEKRRIRRERNKMAAAKCRNRRRELTDTLQAETDQLEDEKSALQ<br>TEIANLLKEKEKLEFILAAHRPACKIPDDLGFPEEMSVASLDLTGGLPEVATPESEEAFT<br>LPLLNDPEPKPSVEPVKSISSMELKTEPFDDFLFPASSRPSGSETARSVPDMDLSGSFYA<br>ADWEPLHSGSLGMGPMATELEPLCTPVVTCTPSCTAYTSSFVFTYPEADSFPSCAAAHRK<br>GSSSNEPSSDSLSSPTLLAL |
| 17 | >sp\|P05412\|AP1_HUMAN Transcription factor AP-1 (Activator protein 1) (AP1) (Proto-oncogene c-jun) (V-jun avian sarcoma virus 17 oncogene homolog) (p39) - Homo sapiens (Human).<br>MTAKMETTFYDDALNASFLPSESGPYGYSNPKILKQSMTLNLADPVGSLKPHLRAKNSDL<br>LTSPDVGLLKLASPELERLIIQSSNGHITTTPTPTQFLCPKNVTDEQEGFAEGFVRALAE<br>LHSQNTLPSVTSAAQPVNGAGMVAPAVASVAGGSGSGGFSASLHSEPPVYANLSNFNPGA<br>LSSGGGAPSYGAAGLAFPAQPQQQQPPHHLPQQMPVQHPRLQALKEEPQTVPEMPGETP<br>PLSPIDMESQERIKAERKRMRNRIAASKCRKRKLERIARLEEKVKTLKAQNSELASTANM<br>LREQVAQLKQKVMNHVNSGCQLMLTQQLQTF |

| | |
|---|---|
| 18 | >sp\|P17275\|JUNB_HUMAN Transcription factor jun-B - Homo sapiens (Human).<br>MCTKMEQPFYHDDSYTATGYGRAPGGLSLHDYKLLKPSLAVNLADPYRSLKAPGARGPGP<br>EGGGGGSYFSGQGSDTGASLKLASSELERLIVPNSNGVITTTPTPPGQYFYPRGGGSGGG<br>AGGAGGGVTEEQEGFADGFVKALDDLHKMNHVTPPNVSLGATGGPPAGPGGVYAGPEPPP<br>VYTNLSSYSPASASSGGAGAAVGTGSSYPTTTISYLPHAPPFAGGHPAQLGLGRGASTFK<br>EEPQTVPEARSRDATPPVSPINMEDQERIKVERKRLRNRLAATKCRKRKLERIARLEDKV<br>KTLKAENAGLSSTAGLLREQVAQLKQKVMTHVSNGCQLLLGVKGHAF |
| 19 | >sp\|P19138\|KC21_HUMAN Casein kinase II, alpha chain (CK II) (EC 2.7.1.37) - Homo sapiens (Human), and Bos taurus (Bovine).<br>MSGPVPSRARVYTDVNTHRPREYWDYESHVVEWGNQDDYQLVRKLGRGKYSEVFEAINIT<br>NNEKVVVKILKPVKKKKIKREIKILENLRGGPNIITLADIVKDPVSRTPALVFEHVNNTD<br>FKQLYQTLTDYDIRFYMYEILKALDYCHSMGIMHRDVKPHNVMIDHEHRKLRLIDWGLAE<br>FYHPGQEYNVRVASRYFKGPELLVDYQMYDYSLDMWSLGCMLASMIFRKEPFFHGHDNYD<br>QLVRIAKVLGTEDLYDYIDKYNIELDPRFNDILGRHSRKRWERFVHSENQHLVSPEALDF<br>LDKLLRYDHQSRLTAREAMEHPYFYTVVKDQARMGSSSMPGGSTPVSSANMMSGISSVPT<br>PSPLGPLAGSPVIAAANPLGMPVPAAAGAQQ |
| 20 | >sp\|P45983\|MK08_HUMAN Mitogen-activated protein kinase 8 (EC 2.7.1.37) (Stress-activated protein kinase JNK1) (c-Jun N-terminal kinase 1) (JNK-46) - Homo sapiens (Human).<br>MSRSKRDNNFYSVEIGDSTFTVLKRYQNLKPIGSGAQGIVCAAYDAILERNVAIKKLSRP<br>FQNQTHAKRAYRELVLMKCVNHKNIIGLLNVFTPQKSLEEFQDVYIVMELMDANLCQVIQ<br>MELDHERMSYLLYQMLCGIKHLHSAGIIHRDLKPSNIVVKSDCTLKILDFGLARTAGTSF<br>MMTPYVVTRYYRAPEVILGMGYKENVDLWSVGCIMGEMVCHKILFPGRDYIDQWNKVIEQ<br>LGTPCPEFMKKLQPTVRTYVENRPKYAGYSFEKLFPDVLFPADSEHNKLKASQARDLLSK<br>MLVIDASKRISVDEALQHPYINVWYDPSEAEAPPPKIPDKQLDEREHTIEEWKELIYKEV<br>MDLEERTKNGVIRGQPSPLGAAVINGSQHPSSSSSVNDVSSMSTDPTLASDTDSSLEAAA<br>GPLGCCR |

**Table S2**

Results of search from nr database for pentameric sequences containing E:D:G:C::2:1:1:1. A key denoting highlights is below. Disintegrin and ADAM proteins are well represented (30 examples), as well as a number of receptors of various types (34 examples). Entries 347 and 348 are the insulin receptor precursor.

KEY:

| | |
|---|---|
| ADAM; disintegrin | (yellow highlight) |
| Zinc | (blue highlight) |
| receptor | (green highlight) |
| Insulin | (yellow highlight) |
| **Calcium** | (blue, bold) |
| **Sodium; Na** | (green, bold) |

**1.** GEECD: 56, >IPI:IPI00288894.1|SWISS-PROT:P78536-1|REFSEQ_NP:NP_003174|ENSEMBL:ENSP00000309968 Tax_Id=9606 Splice isoform A of P78536 ADAM 17 precursor

**2.** GEECD: 64, >IPI:IPI00029606.1|SWISS-PROT:P78536-2|REFSEQ_NP:NP_068604|ENSEMBL:ENSP00000281420 Tax_Id=9606 Splice isoform B of P78536 ADAM 17 precursor

**3.** DECEG: 92, >IPI:IPI00157513.1|REFSEQ_NP:NP_640329|TREMBL:Q96RW4|ENSEMBL:ENSP00000276935 Tax_Id=9606 ADAM-TS related protein 1 isoform 1

**4.** CEDEG: 276, >IPI:IPI00140138.1|REFSEQ_NP:NP_064540|TREMBL:Q96N19|ENSEMBL:ENSP00000310351 Tax_Id=9606 Hypothetical protein FLJ31532

**5.** EDEGC: 276, >IPI:IPI00140138.1|REFSEQ_NP:NP_064540|TREMBL:Q96N19|ENSEMBL:ENSP00000310351 Tax_Id=9606 Hypothetical protein FLJ31532

**6.** EDEGC: 790, >IPI:IPI00215659.1|TREMBL:Q96MX2 Tax_Id=9606 Hypothetical protein FLJ31755

**7.** CEEGD: 944, >IPI:IPI00173394.2|REFSEQ_XP:XP_210139 Tax_Id=9606 similar to hypothetical protein MGC23885

**8.** ECEGD: 2026, >IPI:IPI00232476.1|TREMBL:O75341|ENSEMBL:ENSP00000261737 Tax_Id=9606 BRCA1-associated protein 2

**9.** DGCEE: 2066, >IPI:IPI00097548.1|SWISS-PROT:Q9BZW2|REFSEQ_NP:NP_071889|ENSEMBL:ENSP00000194130 Tax_Id=9606 Solute carrier family 13, member 1

**10.** GCEED: 2248, >IPI:IPI00064885.2|REFSEQ_NP:NP_694563|TREMBL:Q96NJ6|ENSEMBL:ENSP00000320347 Tax_Id=9606 Hypothetical protein FLJ30726

**11.** GEDCE: 2776, >IPI:IPI00332150.1|ENSEMBL:ENSP00000332692 Tax_Id=9606

**12.** CEGDE: 3266, >IPI:IPI00173549.1|TREMBL:Q9UK89;Q9UMS6|REFSEQ_XP:XP_050219|ENSEMBL:ENSP00000306015 Tax_Id=9606 similar to Myopodin protein

**13.** EGCDE: 3368, >IPI:IPI00289489.1|SWISS-PROT:O15230|REFSEQ_NP:NP_005551|TREMBL:O75079;Q8TDF8|ENSEMBL:ENSP00000252999 Tax_Id=9606 Laminin alpha-5 chain precursor

**14.** CEGED: 3828, >IPI:IPI00332170.1|ENSEMBL:ENSP00000332727 Tax_Id=9606

**15.** DCEGE: 3828, >IPI:IPI00332170.1|ENSEMBL:ENSP00000332727 Tax_Id=9606

**16.** DCEEG: 3936, >IPI:IPI00018298.3|SWISS-PROT:P09913|REFSEQ_NP:NP_001538|TREMBL:Q8IZ03|ENSEMBL:ENSP00000238977 Tax_Id=9606 interferon-induced protein with tetratricopeptide repeats 2

**17.** GDECE: 3974, >IPI:IPI00329773.2|SWISS-PROT:Q9NR19|REFSEQ_NP:NP_061147|TREMBL:Q9BYP4;Q96FY7;Q9BYP2;Q9H9U4;Q8N238|ENSEMBL:ENSP00000253382 Tax_Id=9606 Acetyl-coenzyme A synthetase, cytoplasmic

**18.** EGCED: 4522, >IPI:IPI00215830.1|REFSEQ_NP:NP_001557|TREMBL:O15326|ENSEMBL:ENSP00000323547 Tax_Id=9606 Inositol polyphosphate 4-phosphatase type I-beta

**19.** EGCED: 4720, >IPI:IPI00044388.1|REFSEQ_NP:NP_004018|TREMBL:Q96PE3;Q8TC02;Q13187|ENSEMBL:ENSP00000074304 Tax_Id=9606 Inositol polyphosphate 4-phosphatase type I alpha 3

**20.** EDEGC: 4906, >IPI:IPI00015931.1|SWISS-PROT:P17658|REFSEQ_NP:NP_002226|ENSEMBL:ENSP00000280684 Tax_Id=9606 Potassium voltage-gated channel subfamily A member 6

**21.** DECGE: 4952, >IPI:IPI00215890.1|SWISS-PROT:P17026|REFSEQ_NP:NP_008894|ENSEMBL:ENSP00000298299 Tax_Id=9606 zinc finger protein 22 (KOX 15)

**22.** CGEED: 5108, >IPI:IPI00044600.2|SWISS-PROT:Q96PQ0|REFSEQ_NP:NP_065828|ENSEMBL:ENSP00000329124 Tax_Id=9606 VPS10 domain-containing receptor SorCS2 precursor

**23.** GEEDC: 5112, >IPI:IPI00289801.1|TREMBL:Q96Q02;Q9H6R2;Q96H77|ENSEMBL:ENSP00000320183;ENSP00000329529;ENSP00000329417 Tax_Id=9606 Hypothetical protein KIAA1885

**24.** EGEDC: 5124, >IPI:IPI00140833.1|TREMBL:Q96Q04|REFSEQ_XP:XP_055866|ENSEMBL:ENSP00000270238 Tax_Id=9606 Hypothetical protein KIAA1883

**25.** ECEDG: 5240, >IPI:IPI00332253.1|ENSEMBL:ENSP00000332862;ENSP00000333000 Tax_Id=9606

**26.** CGEDE: 5334, >IPI:IPI00332264.1|TREMBL:Q8TBX6|ENSEMBL:ENSP00000253490 Tax_Id=9606 Hypothetical protein

**27.** CEDEG: 5346, >IPI:IPI00332270.1|ENSEMBL:ENSP00000332885 Tax_Id=9606

**28.** GEECD: 5366, >IPI:IPI00289829.1|SWISS-PROT:Q9UKF5-1|REFSEQ_NP:NP_055084|ENSEMBL:ENSP00000302171;ENSP00000330560 Tax_Id=9606 Splice isoform Alpha of Q9UKF5 ADAM 29 precursor

**29.** GEECD: 5368, >IPI:IPI00029112.1|SWISS-PROT:Q9UKF5-2|REFSEQ_NP:NP_068551 Tax_Id=9606 Splice isoform Beta of Q9UKF5 ADAM 29 precursor

**30.** GEECD: 5370, >IPI:IPI00029113.1|SWISS-PROT:Q9UKF5-3|REFSEQ_NP:NP_068552 Tax_Id=9606 Splice isoform Gamma of Q9UKF5 ADAM 29 precursor

**31.** CEDGE: 5984, >IPI:IPI00233595.1|REFSEQ_XP:XP_293565 Tax_Id=9606 similar to chromosome 11 open reading frame2

**32.** GEEDC: 6554, >IPI:IPI00181360.2|TREMBL:Q96QP9 Tax_Id=9606 SDC-SIGN1B type II isoform

**33.** GEEDC: 6576, >IPI:IPI00290006.1|TREMBL:Q96QQ1 Tax_Id=9606 MDC-SIGN1B type I isoform

**34.** GEEDC: 6586, >IPI:IPI00045033.1|TREMBL:Q96QQ5|ENSEMBL:ENSP00000315407 Tax_Id=9606 SDC-SIGN1A type I isoform

**35.** GEEDC: 6590, >IPI:IPI00045034.1|TREMBL:Q96QQ6 Tax_Id=9606 MDC-SIGN1A type IV isoform

**36.** GEEDC: 6594, >IPI:IPI00290007.3|TREMBL:Q96QQ3;Q96QQ7|ENSEMBL:ENSP00000204801 Tax_Id=9606 MDC-SIGN1A type III isoform

**37.** CEEDG: 7190, >IPI:IPI00029778.1|SWISS-PROT:Q12888|REFSEQ_NP:NP_005648|ENSEMBL:ENSP00000263801 Tax_Id=9606 Tumor suppressor p53-binding protein 1

**38.** DEEGC: 7704, >IPI:IPI00234213.1|TREMBL:O75851|ENSEMBL:ENSP00000262089 Tax_Id=9606 WUGSC:H_DJ0751H13.1 protein

**39.** EDCGE: 7704, >IPI:IPI00234213.1|TREMBL:O75851|ENSEMBL:ENSP00000262089 Tax_Id=9606 WUGSC:H_DJ0751H13.1 protein

**40.** CGEED: 7754, >IPI:IPI00183001.2|REFSEQ_XP:XP_096330 Tax_Id=9606 hypothetical protein XP_096330

**41.** CDGEE: 7842, >IPI:IPI00005774.1|REFSEQ_NP:NP_004622|TREMBL:Q9BR78;Q14114|ENSEMBL:ENSP00000321958 Tax_Id=9606 Apolipoprotein E receptor 2 precursor

**42.** EDEGC: 8036, >IPI:IPI00170531.1|REFSEQ_NP:NP_114092|TREMBL:Q96RP8;Q9BYS4|ENSEMBL:ENSP00000221444 Tax_Id=9606 Voltage-gated potassium channel KCNA7

**43.** CGEED: 8192, >IPI:IPI00016859.2|SWISS-PROT:Q92994-1|REFSEQ_NP:NP_001510|ENSEMBL:ENSP00000329029 Tax_Id=9606 Splice isoform 1 of Q92994 Transcription factor IIIB 90 kDa subunit

**44.** CGEED: 8198, >IPI:IPI00157643.1|SWISS-PROT:Q92994-3|REFSEQ_NP:NP_663718 Tax_Id=9606 Splice isoform 3 of Q92994 Transcription factor IIIB 90 kDa subunit

**45.** DECGE: 8800, >IPI:IPI00028450.1|SWISS-PROT:P32418-1|REFSEQ_NP:NP_066920|ENSEMBL:ENSP00000332931 Tax_Id=9606 Splice isoform 1 of P32418 **Sodium**/**calcium** exchanger 1 precursor

**46.** DECGE: 8802, >IPI:IPI00216107.1|SWISS-PROT:P32418-2|TREMBL:Q9UD55;Q14913 Tax_Id=9606 Splice isoform 3 of P32418 **Sodium**/**calcium** exchanger 1 precursor

**47.** DECGE: 8808, >IPI:IPI00216108.1|SWISS-PROT:P32418-3 Tax_Id=9606 Splice isoform 7 of P32418 **Sodium**/**calcium** exchanger 1 precursor

**48.** DECGE: 8812, >IPI:IPI00216109.1|SWISS-PROT:P32418-4 Tax_Id=9606 Splice isoform 10 of P32418 **Sodium**/**calcium** exchanger 1 precursor

**49.** CEEGD: 9158, >IPI:IPI00290465.1|TREMBL:Q8TAN8 Tax_Id=9606 Dynactin 4

**50.** EGECD: 9324, >IPI:IPI00141177.1|REFSEQ_XP:XP_115639 Tax_Id=9606 similar to leukocyte immunoglobulin-like receptor, subfamily B (with TM and ITIM domains), member 6

**51.** CEDGE: 9356, >IPI:IPI00021537.2|SWISS-PROT:Q9NZT2-1|REFSEQ_NP:NP_031372|ENSEMBL:ENSP00000290291 Tax_Id=9606 Splice isoform 1 of Q9NZT2 Opioid growth factor receptor

**52.** CEDGE: 9358, >IPI:IPI00216130.1|SWISS-PROT:Q9NZT2-2 Tax_Id=9606 Splice isoform 2 of Q9NZT2 Opioid growth factor receptor

**53.** EDGEC: 9416, >IPI:IPI00170891.1|REFSEQ_NP:NP_683702;NP_683703|TREMBL:Q8TAS8 Tax_Id=9606 Oxysterol binding protein-like 9

**54.** CGEED: 9420, >IPI:IPI00152108.1|REFSEQ_NP:NP_036300|TREMBL:Q8TB29|ENSEMBL:ENSP00000235299 Tax_Id=9606 F-box only protein 2

**55.** EDGEC: 9498, >IPI:IPI00290550.1|REFSEQ_NP:NP_683705|TREMBL:Q96SK4 Tax_Id=9606 Hypothetical protein FLJ14801

**56.** GCEED: 9588, >IPI:IPI00178375.1|TREMBL:Q8TB50|ENSEMBL:ENSP00000313070 Tax_Id=9606 Similar to KIAA0433 protein

**57.** EEDGC: 9656, >IPI:IPI00014211.1|SWISS-PROT:Q07011|REFSEQ_NP:NP_001552|ENSEMBL:ENSP00000054670 Tax_Id=9606 Tumor necrosis factor receptor superfamily member 9 precursor

**58.** DCGEE: 10064, >IPI:IPI00216170.1|SWISS-PROT:P10159|REFSEQ_NP:NP_001961|ENSEMBL:ENSP00000254843 Tax_Id=9606 eukaryotic translation initiation factor 5A

**59.** ECGDE: 10516, >IPI:IPI00235181.1|REFSEQ_XP:XP_305828 Tax_Id=9606 hypothetical protein XP_305828

**60.** CEGED: 11724, >IPI:IPI00011836.1|SWISS-PROT:Q13107-1|REFSEQ_NP:NP_003354|ENSEMBL:ENSP00000265560 Tax_Id=9606 Splice isoform UNPEL of Q13107 Ubiquitin carboxyl-terminal hydrolase 4

**61.** CEGED: 11726, >IPI:IPI00216257.1|SWISS-PROT:Q13107-2 Tax_Id=9606 Splice isoform UNPES of Q13107 Ubiquitin carboxyl-terminal hydrolase 4

**62.** CEGED: 12024, >IPI:IPI00102864.1|REFSEQ_NP:NP_000180|TREMBL:Q8WU87|ENSEMBL:ENSP00000290573 Tax_Id=9606 Hexokinase 2

**63.** DEGEC: 12572, >IPI:IPI00235759.1|REFSEQ_XP:XP_305957 Tax_Id=9606 similar to olfactory receptor

**64.** GDECE: 12766, >IPI:IPI00027107.3|SWISS-PROT:P49411|REFSEQ_NP:NP_003312|ENSEMBL:ENSP00000322439 Tax_Id=9606 Tu translation elongation factor, mitochondrial

**65.** ECDGE: 12864, >IPI:IPI00046309.1|REFSEQ_XP:XP_066534|ENSEMBL:ENSP00000289280 Tax_Id=9606 similar to Diacylglycerol kinase, delta (Diglyceride kinase) (DGK-delta) (DAG kinase delta) (130 kDa diacylglycerol kinase)

**66.** ECEGD: 13184, >IPI:IPI00152462.1|REFSEQ_NP:NP_060009|TREMBL:O15437;Q9UEM5;O43326;Q9UG35;Q8TD57;Q9UEM3;O00437|ENSEMBL:ENSP00000261383 Tax_Id=9606 Axonemal heavy chain dynein type 3

**67.** EEGCD: 13620, >IPI:IPI00031015.1|SWISS-PROT:Q14493|REFSEQ_NP:NP_006518|ENSEMBL:ENSP00000316490 Tax_Id=9606 Histone RNA hairpin-binding protein

**68.** CEDGE: 13680, >IPI:IPI00178788.2|ENSEMBL:ENSP00000318933 Tax_Id=9606

**69.** DCGEE: 13680, >IPI:IPI00178788.2|ENSEMBL:ENSP00000318933 Tax_Id=9606

**70.** EDCGE: 13680, >IPI:IPI00178788.2|ENSEMBL:ENSP00000318933 Tax_Id=9606

**71.** EDGEC: 13680, >IPI:IPI00178788.2|ENSEMBL:ENSP00000318933 Tax_Id=9606

**72.** ECEDG: 13890, >IPI:IPI00178829.1|ENSEMBL:ENSP00000276891 Tax_Id=9606

**73.** GEECD: 14396, >IPI:IPI00019173.2|SWISS-PROT:Q99965-1|REFSEQ_NP:NP_001455|ENSEMBL:ENSP00000265708 Tax_Id=9606 Splice isoform 1 of Q99965 ADAM 2 precursor

**74.** GEECD: 14398, >IPI:IPI00216374.1|SWISS-PROT:Q99965-2 Tax_Id=9606 Splice isoform 2 of Q99965 ADAM 2 precursor

**75.** DEEGC: 15648, >IPI:IPI00178940.2|ENSEMBL:ENSP00000320248 Tax_Id=9606

**76.** EDEGC: 16138, >IPI:IPI00157767.1|TREMBL:Q8TEJ1 Tax_Id=9606 FLJ00206 protein

**77.** DCGEE: 16788, >IPI:IPI00152847.1|REFSEQ_NP:NP_783165|TREMBL:Q8TEU8|ENSEMBL:ENSP00000311184 Tax_Id=9606 Multivalent protease inhibitor protein

**78.** EDCGE: 16788, >IPI:IPI00152847.1|REFSEQ_NP:NP_783165|TREMBL:Q8TEU8|ENSEMBL:ENSP00000311184 Tax_Id=9606 Multivalent protease inhibitor protein

**79.** CEGED: 16872, >IPI:IPI00304849.3|REFSEQ_NP:NP_079406|TREMBL:Q8WU37;Q96EH2|ENSEMBL:ENSP00000298648 Tax_Id=9606 hypothetical protein FLJ22761

**80.** DCEGE: 16872, >IPI:IPI00304849.3|REFSEQ_NP:NP_079406|TREMBL:Q8WU37;Q96EH2|ENSEMBL:ENSP00000298648 Tax_Id=9606 hypothetical protein FLJ22761

**81.** EDCEG: 17896, >IPI:IPI00292059.1|SWISS-PROT:P49790|REFSEQ_NP:NP_005115|ENSEMBL:ENSP00000262077 Tax_Id=9606 Nuclear pore complex protein Nup153

**82.** CEGED: 17902, >IPI:IPI00221325.2|SWISS-PROT:P49792|REFSEQ_NP:NP_006258|TREMBL:Q13073;Q13074|ENSEMBL:ENSP00000283195;ENSP00000315836 Tax_Id=9606 Ran-binding protein 2

**83.** DCEGE: 17902, >IPI:IPI00221325.2|SWISS-PROT:P49792|REFSEQ_NP:NP_006258|TREMBL:Q13073;Q13074|ENSEMBL:ENSP00000283195;ENSP00000315836 Tax_Id=9606 Ran-binding protein 2

**84.** GCEED: 18972, >IPI:IPI00237376.1|REFSEQ_XP:XP_295599 Tax_Id=9606 hypothetical protein XP_295599

**85.** CEGED: 19342, >IPI:IPI00145406.3|TREMBL:O14715|REFSEQ_XP:XP_300391|ENSEMBL:ENSP00000306637 Tax_Id=9606 similar to RAN-binding protein 2-like 1 isoform 1

**86.** DCEGE: 19342, >IPI:IPI00145406.3|TREMBL:O14715|REFSEQ_XP:XP_300391|ENSEMBL:ENSP00000306637 Tax_Id=9606 similar to RAN-binding protein 2-like 1 isoform 1

**87.** CEDGE: 19528, >IPI:IPI00332725.1|ENSEMBL:ENSP00000327776 Tax_Id=9606

**88.** DCGEE: 19528, >IPI:IPI00332725.1|ENSEMBL:ENSP00000327776 Tax_Id=9606

**89.** EDCGE: 19528, >IPI:IPI00332725.1|ENSEMBL:ENSP00000327776 Tax_Id=9606

**90.** EDGEC: 19528, >IPI:IPI00332725.1|ENSEMBL:ENSP00000327776 Tax_Id=9606

**91.** DEGEC: 20088, >IPI:IPI00297629.2|SWISS-PROT:O76001|TREMBL:Q96R15|REFSEQ_XP:XP_212594;XP_305975|ENSEMBL:ENSP00000259884 Tax_Id=9606 similar to Olfactory receptor 2J3 (Olfactory receptor 6-6) (OR6-6) (Hs6M1-3)

**92.** GEECD: 20720, >IPI:IPI00069817.2|SWISS-PROT:Q9UIG0-1|REFSEQ_NP:NP_115784|ENSEMBL:ENSP00000265756 Tax_Id=9606 Splice isoform 1 of Q9UIG0 Bromodomain adjacent to zinc finger domain protein 1B

**93.** GEECD: 20722, >IPI:IPI00216695.1|SWISS-PROT:Q9UIG0-2|TREMBL:Q86UJ6 Tax_Id=9606 Splice isoform 2 of Q9UIG0 Bromodomain adjacent to zinc finger domain protein 1B

**94.** DGCEE: 20962, >IPI:IPI00103925.1|TREMBL:Q8WZA9|REFSEQ_XP:XP_065026|ENSEMBL:ENSP00000300809 Tax_Id=9606 FKSG27

**95.** DECEG: 21696, >IPI:IPI00328113.2|SWISS-PROT:P35555|REFSEQ_NP:NP_000129|TREMBL:Q15972;Q9NP01|ENSEMBL:ENSP00000325527 Tax_Id=9606 Fibrillin 1 precursor

**96.** EDEGC: 21832, >IPI:IPI00216878.1|REFSEQ_NP:NP_079174|TREMBL:Q9H5Z2;Q8IV53;Q8NDB1|ENSEMBL:ENSP00000264083 Tax_Id=9606 Hypothetical protein FLJ22757

**97.** DGCEE: 22118, >IPI:IPI00238075.1|REFSEQ_XP:XP_295732 Tax_Id=9606 hypothetical protein XP_295732

**98.** EDGCE: 22232, >IPI:IPI00171453.1|SWISS-PROT:Q9NVP4-1|ENSEMBL:ENSP00000287356 Tax_Id=9606 Splice isoform 1 of Q9NVP4 Protein c20orf12

**99.** EDGCE: 22234, >IPI:IPI00216942.1|SWISS-PROT:Q9NVP4-2|REFSEQ_NP:NP_060622|ENSEMBL:ENSP00000262547 Tax_Id=9606 Splice isoform 2 of Q9NVP4 Protein c20orf12

**100.** DEECG: 22460, >IPI:IPI00216963.1|SWISS-PROT:O15519-9 Tax_Id=9606 Splice isoform 9 of O15519 CASP8 and FADD-like apoptosis regulator precursor

**101.** CEGED: 22538, >IPI:IPI00302502.2|TREMBL:Q9H5Y9|ENSEMBL:ENSP00000287067 Tax_Id=9606 Hypothetical protein FLJ22761

**102.** DCEGE: 22538, >IPI:IPI00302502.2|TREMBL:Q9H5Y9|ENSEMBL:ENSP00000287067 Tax_Id=9606 Hypothetical protein FLJ22761

**103.** EGEDC: 22590, >IPI:IPI00332867.1|ENSEMBL:ENSP00000329165 Tax_Id=9606

**104.** ECEDG: 22634, >IPI:IPI00292859.1|SWISS-PROT:P20023-1|REFSEQ_NP:NP_001868|ENSEMBL:ENSP00000326149 Tax_Id=9606 Splice isoform A of P20023 Complement receptor type 2 precursor

**105.** ECEDG: 22636, >IPI:IPI00216985.1|SWISS-PROT:P20023-2|TREMBL:Q9H2B3 Tax_Id=9606 Splice isoform B of P20023 Complement receptor type 2 precursor

**106.** ECEDG: 22638, >IPI:IPI00216986.1|SWISS-PROT:P20023-3 Tax_Id=9606 Splice isoform C of P20023 Complement receptor type 2 precursor

**107.** ECEDG: 22640, >IPI:IPI00216987.1|SWISS-PROT:P20023-4 Tax_Id=9606 Splice isoform D of P20023 Complement receptor type 2 precursor

**108.** CEGDE: 22700, >IPI:IPI00007956.1|REFSEQ_NP:NP_006463|TREMBL:Q9BXG9;Q9H3M7;Q16226|ENSEMBL:ENSP00000235941 Tax_Id=9606 Brain-expressed HHCPA78 homolog VDUP1

**109.** GEDCE: 22726, >IPI:IPI00332886.1|ENSEMBL:ENSP00000329193 Tax_Id=9606

**110.** CEDGE: 23008, >IPI:IPI00332906.1|ENSEMBL:ENSP00000327936 Tax_Id=9606

**111.** DCGEE: 23008, >IPI:IPI00332906.1|ENSEMBL:ENSP00000327936 Tax_Id=9606

**112.** EDGEC: 23008, >IPI:IPI00332906.1|ENSEMBL:ENSP00000327936 Tax_Id=9606

**113.** GDECE: 23330, >IPI:IPI00238420.1|REFSEQ_XP:XP_297070 Tax_Id=9606 hypothetical protein XP_297070

**114.** EDCEG: 23334, >IPI:IPI00292956.1|TREMBL:Q9NTD6;Q96AL1;Q9P2L9|REFSEQ_XP:XP_051146|ENSEMBL:ENSP00000040738 Tax_Id=9606 KIAA1327 protein

**115.** EECDG: 23334, >IPI:IPI00292956.1|TREMBL:Q9NTD6;Q96AL1;Q9P2L9|REFSEQ_XP:XP_051146|ENSEMBL:ENSP00000040738 Tax_Id=9606 KIAA1327 protein

**116.** EDEGC: 23548, >IPI:IPI00157942.2|REFSEQ_XP:XP_170664|ENSEMBL:ENSP00000329798 Tax_Id=9606 similar to pecanex-like 3

**117.** EGEDC: 23880, >IPI:IPI00217197.1|TREMBL:Q8IW93|ENSEMBL:ENSP00000270747 Tax_Id=9606 Similar to hypothetical protein FLJ33962

**118.** GDCEE: 24742, >IPI:IPI00019004.1|REFSEQ_NP:NP_003253|TREMBL:Q99442|ENSEMBL:ENSP00000009363;ENSP00000310265 Tax_Id=9606 TRANSLOCATIONAL protein-1

**119.** ECDGE: 25188, >IPI:IPI00020557.1|SWISS-PROT:Q07954|REFSEQ_NP:NP_002323|TREMBL:Q86SW0|ENSEMBL:ENSP00000243077 Tax_Id=9606 Low-density lipoprotein receptor-related protein 1 precursor

**120.** EDECG: 25296, >IPI:IPI00217435.1|REFSEQ_NP:NP_766638|TREMBL:Q8IWY4|ENSEMBL:ENSP00000290460 Tax_Id=9606 Signal peptide-CUB-EGF-like domain containing protein 1

**121.** CEEGD: 25570, >IPI:IPI00003443.2|SWISS-PROT:Q16666-1|ENSEMBL:ENSP00000319280 Tax_Id=9606 Splice isoform 1 of Q16666 Gamma-interferon-inducible protein Ifi-16

**122.** CEEGD: 25572, >IPI:IPI00217474.1|SWISS-PROT:Q16666-2|REFSEQ_NP:NP_005522|TREMBL:Q96AJ5;Q8NEQ7|ENSEMBL:ENSP00000295808 Tax_Id=9606 Splice isoform 2 of Q16666 Gamma-interferon-inducible protein Ifi-16

**123.** CEEGD: 25576, >IPI:IPI00217475.1|SWISS-PROT:Q16666-3 Tax_Id=9606 Splice isoform 3 of Q16666 Gamma-interferon-inducible protein Ifi-16

**124.** DEECG: 25998, >IPI:IPI00293293.1|SWISS-PROT:Q99523 Tax_Id=9606 Sortilin precursor

**125.** CEGED: 27810, >IPI:IPI00293568.1|REFSEQ_NP:NP_005045|TREMBL:Q99666|ENSEMBL:ENSP00000016946;ENSP00000330842 Tax_Id=9606 Sperm membrane protein BS-63

**126.** DCEGE: 27810, >IPI:IPI00293568.1|REFSEQ_NP:NP_005045|TREMBL:Q99666|ENSEMBL:ENSP00000016946;ENSP00000330842 Tax_Id=9606 Sperm membrane protein BS-63

**127.** EEGDC: 27922, >IPI:IPI00333139.1|SWISS-PROT:Q99683|REFSEQ_NP:NP_005914|ENSEMBL:ENSP00000265606 Tax_Id=9606 Mitogen-activated protein kinase kinase kinase 5

**128.** EGDCE: 27922, >IPI:IPI00333139.1|SWISS-PROT:Q99683|REFSEQ_NP:NP_005914|ENSEMBL:ENSP00000265606 Tax_Id=9606 Mitogen-activated protein kinase kinase kinase 5

**129.** GEECD: 28000, >IPI:IPI00333140.1|REFSEQ_NP:NP_620711|TREMBL:Q8IYT0;Q9UDM2;Q9NTF1;Q8TB42;Q8NFT8|ENSEMBL:ENSP00000309867 Tax_Id=9606 Similar to delta-notch-like EGF repeat-containing transmembrane

**130.** DEECG: 28068, >IPI:IPI00217882.1|REFSEQ_NP:NP_002950|TREMBL:Q8IZ49|ENSEMBL:ENSP00000256637 Tax_Id=9606 Sortilin 1

**131.** DCGEE: 28478, >IPI:IPI00239974.1|REFSEQ_XP:XP_290186|ENSEMBL:ENSP00000329807 Tax_Id=9606 similar to eukaryotic initiation factor 5A

**132.** ECGED: 28644, >IPI:IPI00031422.3|SWISS-PROT:Q14524|REFSEQ_NP:NP_000326|TREMBL:Q8WWN5;Q8IZC9;Q8WTQ6;Q96J69;Q86UR3;Q8WTS0|ENSEMBL:ENSP00000328968 Tax_Id=9606 Cardiac **sodium** channel alpha subunit **Na**v1.5

**133.** DCGEE: 29618, >IPI:IPI00218084.2|REFSEQ_XP:XP_084467|ENSEMBL:ENSP00000331000 Tax_Id=9606 similar to eukaryotic initiation factor 5A

**134.** CEDGE: 31564, >IPI:IPI00022608.1|SWISS-PROT:Q92673|REFSEQ_NP:NP_003096|ENSEMBL:ENSP00000260197 Tax_Id=9606 Sortilin-related receptor precursor

**135.** ECEGD: 31666, >IPI:IPI00240961.1|REFSEQ_XP:XP_302387|ENSEMBL:ENSP00000333778 Tax_Id=9606 similar to Multifunctional protein ADE2

**136.** GDECE: 33070, >IPI:IPI00294630.1|REFSEQ_NP:NP_644803|ENSEMBL:ENSP00000321387 Tax_Id=9606 acetyl-CoA synthetase 2 isoform b

**137.** DGECE: 33506, >IPI:IPI00163901.1|TREMBL:Q9Y3V7;Q8IUI0;Q8IUI1|ENSEMBL:ENSP00000295761 Tax_Id=9606 Hypothetical protein

**138.** GECED: 33506, >IPI:IPI00163901.1|TREMBL:Q9Y3V7;Q8IUI0;Q8IUI1|ENSEMBL:ENSP00000295761 Tax_Id=9606 Hypothetical protein

**139.** ECDEG: 33580, >IPI:IPI00028987.1|REFSEQ_NP:NP_068506|TREMBL:O95425;Q9H1R7;O60611|ENSEMBL:ENSP00000263057 Tax_Id=9606 Archvillin

**140.** CEDGE: 33702, >IPI:IPI00333400.1|ENSEMBL:ENSP00000329881 Tax_Id=9606

**141.** CDGEE: 34036, >IPI:IPI00241624.1|REFSEQ_XP:XP_297753 Tax_Id=9606 hypothetical protein XP_297753

**142.** ECEGD: 34356, >IPI:IPI00294839.1|SWISS-PROT:Q9Y4K0|REFSEQ_NP:NP_002309|ENSEMBL:ENSP00000256397 Tax_Id=9606 Lysyl oxidase homolog 2 precursor

**143.** CDEGE: 34614, >IPI:IPI00241780.1|REFSEQ_XP:XP_302541 Tax_Id=9606 similar to meningioma-expressed antigen 11

**144.** EEGCD: 34738, >IPI:IPI00333423.1|ENSEMBL:ENSP00000329913 Tax_Id=9606

**145.** DCEGE: 34770, >IPI:IPI00011227.1|SWISS-PROT:P21918|REFSEQ_NP:NP_000789|TREMBL:Q8NEQ8|ENSEMBL:ENSP00000306129 Tax_Id=9606 D(1B) dopamine receptor

**146.** DCGEE: 34788, >IPI:IPI00020083.1|SWISS-PROT:Q15878-1|TREMBL:Q9NY05;Q9UN68;Q9NYZ6|ENSEMBL:ENSP00000252150;ENSP00000252151 Tax_Id=9606 Splice isoform Alpha-1E-3 of Q15878 Voltage-dependent R-type **calcium** channel alpha-1E subunit

**147.** DCGEE: 34794, >IPI:IPI00218338.1|SWISS-PROT:Q15878-2|REFSEQ_NP:NP_000712|ENSEMBL:ENSP00000308642 Tax_Id=9606 Splice isoform Alpha-1E-1 of Q15878 Voltage-dependent R-type **calcium** channel alpha-1E subunit

**148.** DECGE: 35582, >IPI:IPI00004957.1|SWISS-PROT:Q9Y5C1|REFSEQ_NP:NP_055310|ENSEMBL:ENSP00000255208 Tax_Id=9606 Angiopoietin-related protein 3 precursor

**149.** EDGCE: 35928, >IPI:IPI00002493.1|TREMBL:Q9Y5J4|ENSEMBL:ENSP00000321781 Tax_Id=9606 Pyrroline 5-carboxylate reductase isoform

**150.** ECEGD: 37020, >IPI:IPI00242598.1|REFSEQ_XP:XP_299197 Tax_Id=9606 hypothetical protein XP_299197

**151.** EDGEC: 37310, >IPI:IPI00077853.1|SWISS-PROT:Q96SU4-1|REFSEQ_NP:NP_078862|ENSEMBL:ENSP00000303944 Tax_Id=9606 Splice isoform 1 of Q96SU4 Oxysterol binding protein-related protein 9

**152.** EDGEC: 37314, >IPI:IPI00218449.1|SWISS-PROT:Q96SU4-2|REFSEQ_NP:NP_683706 Tax_Id=9606 Splice isoform 2 of Q96SU4 Oxysterol binding protein-related protein 9

**153.** ECEDG: 37612, >IPI:IPI00016698.1|SWISS-PROT:Q9UEU5|REFSEQ_NP:NP_036328 Tax_Id=9606 GAGE-8 protein

**154.** GEDCE: 37860, >IPI:IPI00021968.1|SWISS-PROT:Q9Y6Q6|REFSEQ_NP:NP_003830|ENSEMBL:ENSP00000269485 Tax_Id=9606 Tumor necrosis factor receptor superfamily member 11A precursor

**155.** GDCEE: 37890, >IPI:IPI00098997.1|REFSEQ_NP:NP_003750|TREMBL:O15153;Q9Y6R1;Q9H262;Q9UIC0;Q9UIC1|ENSEMBL:ENSP00000264485 Tax_Id=9606 Pancreas **sodium** bicarbonate cotransporter

**156.** GDCEE: 37894, >IPI:IPI00002561.1|TREMBL:Q9Y6R3 Tax_Id=9606 Electrogenic **Na**+ bicarbonate cotransporter

**157.** CGDEE: 38394, >IPI:IPI00069309.1|REFSEQ_NP:NP_690852|TREMBL:Q8N0T9;Q8WV43;Q96GJ2;Q9UFC0|ENSEMBL:ENSP00000292616 Tax_Id=9606 Hypothetical protein

**158.** EDCGE: 38398, >IPI:IPI00004623.1|TREMBL:Q9UFC1|ENSEMBL:ENSP00000307039 Tax_Id=9606 Hypothetical protein

**159.** EDCEG: 38940, >IPI:IPI00243356.1|REFSEQ_XP:XP_304057 Tax_Id=9606 hypothetical protein XP_304057

**160.** GEDCE: 38940, >IPI:IPI00243356.1|REFSEQ_XP:XP_304057 Tax_Id=9606 hypothetical protein XP_304057

**161.** GEEDC: 39992, >IPI:IPI00104867.1|ENSEMBL:ENSP00000301357 Tax_Id=9606

**162.** CDEEG: 40410, >IPI:IPI00159268.1|REFSEQ_XP:XP_172950 Tax_Id=9606 hypothetical protein XP_172950

**163.** EDEGC: 40844, >IPI:IPI00243959.1|REFSEQ_XP:XP_292142 Tax_Id=9606 similar to bromodomain containing 7

**164.** EDCEG: 40916, >IPI:IPI00243988.1|REFSEQ_XP:XP_304157 Tax_Id=9606 hypothetical protein XP_304157

**165.** EGDCE: 41000, >IPI:IPI00002284.1|SWISS-PROT:Q9UGP5|REFSEQ_NP:NP_037406|TREMBL:Q9HAJ3|ENSEMBL:ENSP00000299206 Tax_Id=9606 DNA polymerase lambda

**166.** CEEGD: 41850, >IPI:IPI00244195.1|REFSEQ_XP:XP_299401 Tax_Id=9606 hypothetical protein XP_299401

**167.** CEDGE: 42152, >IPI:IPI00025276.1|SWISS-PROT:P22105-1|REFSEQ_NP:NP_061978|TREMBL:Q9UC13;Q9UC12;Q9UC11;Q8N4R1;Q9UE34;Q9NPK7;O95681;O95682;Q9Y464|ENSEMBL:ENSP00000211407;ENSP00000299669;ENSP00000329801 Tax_Id=9606 Splice isoform XB of P22105 Tenascin-X precursor

**168.** DCGEE: 42152, >IPI:IPI00025276.1|SWISS-PROT:P22105-1|REFSEQ_NP:NP_061978|TREMBL:Q9UC13;Q9UC12;Q9UC11;Q8N4R1;Q9UE34;Q9NPK7;O95681;O95682;Q9Y464|ENSEMBL:ENSP00000211407;ENSP00000299669;ENSP00000329801 Tax_Id=9606 Splice isoform XB of P22105 Tenascin-X precursor

**169.** EDCGE: 42152, >IPI:IPI00025276.1|SWISS-PROT:P22105-1|REFSEQ_NP:NP_061978|TREMBL:Q9UC13;Q9UC12;Q9UC11;Q8N4R1;Q9UE34;Q9NPK7;O95681;O95682;Q9Y464|ENSEMBL:ENSP00000211407;ENSP00000299669;ENSP00000329801 Tax_Id=9606 Splice isoform XB of P22105 Tenascin-X precursor

**170.** EDGEC: 42152, >IPI:IPI00025276.1|SWISS-PROT:P22105-1|REFSEQ_NP:NP_061978|TREMBL:Q9UC13;Q9UC12;Q9UC11;Q8N4R1;Q9UE34;Q9NPK7;O95681;O95682;Q9Y464|ENSEMBL:ENSP00000211407;ENSP00000299669;ENSP00000329801 Tax_Id=9606 Splice isoform XB of P22105 Tenascin-X precursor

**171.** DCGEE: 42156, >IPI:IPI00218639.1|SWISS-PROT:P22105-2|REFSEQ_NP:NP_115859|ENSEMBL:ENSP00000333864;ENSP00000330575 Tax_Id=9606 Splice isoform XB-short of P22105 Tenascin-X precursor

**172.** DECGE: 42464, >IPI:IPI00328412.1|REFSEQ_NP:NP_115823|TREMBL:Q86SJ5|ENSEMBL:ENSP00000270509 Tax_Id=9606 Fibrillin-3 short form precursor transcript variant 1

**173.** EDECG: 42464, >IPI:IPI00328412.1|REFSEQ_NP:NP_115823|TREMBL:Q86SJ5|ENSEMBL:ENSP00000270509 Tax_Id=9606 Fibrillin-3 short form precursor transcript variant 1

**174.** GEECD: 43964, >IPI:IPI00079482.1|SWISS-PROT:Q9BZ11-1|REFSEQ_NP:NP_079496|TREMBL:Q8N0W6;Q8N6B9|ENSEMBL:ENSP00000278788 Tax_Id=9606 Splice isoform 1 of Q9BZ11 ADAM 33 precursor

**175.** GEECD: 43974, >IPI:IPI00218715.1|SWISS-PROT:Q9BZ11-2|REFSEQ_NP:NP_694882|ENSEMBL:ENSP00000322550 Tax_Id=9606 Splice isoform 2 of Q9BZ11 ADAM 33 precursor

**176.** GEECD: 44974, >IPI:IPI00181689.1|SWISS-PROT:O43184-2|REFSEQ_NP:NP_067673|ENSEMBL:ENSP00000310004 Tax_Id=9606 Splice isoform 12S of O43184 ADAM 12 precursor

**177.** EEGDC: 45106, >IPI:IPI00245008.1|REFSEQ_XP:XP_304348 Tax_Id=9606 hypothetical protein XP_304348

**178.** EGCDE: 45166, >IPI:IPI00245026.1|REFSEQ_XP:XP_292354 Tax_Id=9606 similar to prohibitin

**179.** EEGCD: 45344, >IPI:IPI00333835.1|ENSEMBL:ENSP00000308853 Tax_Id=9606

**180.** GDCEE: 45448, >IPI:IPI00166570.2|TREMBL:Q8N3L3;Q8N3S2;Q86T52|ENSEMBL:ENSP00000321883 Tax_Id=9606 Hypothetical protein DKFZp451F022

**181.** EGDEC: 45712, >IPI:IPI00166602.1|TREMBL:Q8N3R5;Q8N1M9 Tax_Id=9606 Hypothetical protein

**182.** DCEEG: 45754, >IPI:IPI00296542.1|SWISS-PROT:P16581|REFSEQ_NP:NP_000441|ENSEMBL:ENSP00000066315 Tax_Id=9606 E-selectin precursor

**183.** CDEGE: 46368, >IPI:IPI00181793.1|SWISS-PROT:P10911|REFSEQ_NP:NP_005360|TREMBL:Q8IUF4;Q9UJB3;Q8IUF3|ENSEMBL:ENSP00000218094 Tax_Id=9606 Hypothetical protein

**184.** DEGEC: 46368, >IPI:IPI00181793.1|SWISS-PROT:P10911|REFSEQ_NP:NP_005360|TREMBL:Q8IUF4;Q9UJB3;Q8IUF3|ENSEMBL:ENSP00000218094 Tax_Id=9606 Hypothetical protein

**185.** CDGEE: 46970, >IPI:IPI00328785.1|TREMBL:Q86V27 Tax_Id=9606 Similar to low density lipoprotein receptor-related protein 8, apolipoprotein e receptor

**186.** CEDEG: 47272, >IPI:IPI00166717.1|TREMBL:Q8N4K6 Tax_Id=9606 Similar to chromosome 11 hypothetical protein ORF4

**187.** EDEGC: 47272, >IPI:IPI00166717.1|TREMBL:Q8N4K6 Tax_Id=9606 Similar to chromosome 11 hypothetical protein ORF4

**188.** CEEDG: 47288, >IPI:IPI00245636.1|REFSEQ_XP:XP_292482|ENSEMBL:ENSP00000333450 Tax_Id=9606 similar to U3 small nucleolar ribonucleoprotein protein MPP10 (M phase phosphoprotein 10)

**189.** CGEED: 47416, >IPI:IPI00007087.1|SWISS-PROT:Q9UK22 Tax_Id=9606 F-box only protein 2

**190.** CEEGD: 47622, >IPI:IPI00008210.1|REFSEQ_NP:NP_057305|TREMBL:Q9NSJ5;Q9UJW0|ENSEMBL:ENSP00000255263 Tax_Id=9606 Dynactin P62 subunit

**191.** ECEDG: 47948, >IPI:IPI00166789.1|REFSEQ_NP:NP_690875|TREMBL:Q8NCX3;Q8N555;Q86WP9|ENSEMBL:ENSP00000278060 Tax_Id=9606 Similar to chromosome 20 open reading frame 16

**192.** EGEDC: 48062, >IPI:IPI00296830.1|TREMBL:Q8N4X3|REFSEQ_XP:XP_290932|ENSEMBL:ENSP00000303758 Tax_Id=9606 Similar to FLJ00226 protein

**193.** CGEDE: 49572, >IPI:IPI00002545.1|REFSEQ_NP:NP_037496|TREMBL:Q9H0A4;Q9UL41 Tax_Id=9606 Paraneoplastic neuronal antigen MA3

**194.** EDCGE: 49572, >IPI:IPI00002545.1|REFSEQ_NP:NP_037496|TREMBL:Q9H0A4;Q9UL41 Tax_Id=9606 Paraneoplastic neuronal antigen MA3

**195.** GCEED: 49892, >IPI:IPI00297089.1|SWISS-PROT:Q13023|REFSEQ_NP:NP_004265|ENSEMBL:ENSP00000280979 Tax_Id=9606 A-kinase anchor protein 6

**196.** ECEDG: 50158, >IPI:IPI00011526.1|SWISS-PROT:Q13065|REFSEQ_NP:NP_001459|TREMBL:Q8IYC5|ENSEMBL:ENSP00000217878 Tax_Id=9606 GAGE-1 protein

**197.** ECEDG: 50160, >IPI:IPI00011527.1|SWISS-PROT:Q13066|REFSEQ_NP:NP_001463|ENSEMBL:ENSP00000327927 Tax_Id=9606 GAGE-2 protein

**198.** ECEDG: 50166, >IPI:IPI00011529.1|SWISS-PROT:Q13067 Tax_Id=9606 GAGE-3 protein

**199.** ECEDG: 50176, >IPI:IPI00011530.1|SWISS-PROT:Q13068|REFSEQ_NP:NP_001465|ENSEMBL:ENSP00000309195;ENSP00000331882;ENSP00000333538 Tax_Id=9606 GAGE-4 protein

**200.** ECEDG: 50180, >IPI:IPI00011531.1|SWISS-PROT:Q13069|REFSEQ_NP:NP_001466 Tax_Id=9606 GAGE-5 protein

**201.** ECEDG: 50232, >IPI:IPI00011545.1|SWISS-PROT:Q13070|REFSEQ_NP:NP_001467 Tax_Id=9606 GAGE-6 protein

**202.** DGEEC: 50486, >IPI:IPI00246380.1|REFSEQ_XP:XP_299867 Tax_Id=9606 hypothetical protein XP_299867

**203.** EDEGC: 50594, >IPI:IPI00329085.1|REFSEQ_NP:NP_115582|TREMBL:Q8NF42;Q86WI3;Q8TEE2;Q969L7|ENSEMBL:ENSP00000262510;ENSP00000308886 Tax_Id=9606 NOD27

**204.** DCEEG: 50676, >IPI:IPI00166972.1|TREMBL:Q8IY05;Q9ULD9;Q9UFL4;Q9Y5A1|REFSEQ_XP:XP_114432|ENSEMBL:ENSP00000307746 Tax_Id=9606 KIAA1281 protein

**205.** CEEGD: 50882, >IPI:IPI00297250.1|TREMBL:O60290|REFSEQ_XP:XP_044213|ENSEMBL:ENSP00000223210 Tax_Id=9606 Hypothetical protein KIAA0543

**206.** CEEDG: 51488,
>IPI:IPI00329142.1|TREMBL:Q86X53;Q9P063|REFSEQ_XP:XP_291282|ENSEMBL:ENSP00000
262109 Tax_Id=9606 Hypothetical protein

**207.** EDEGC: 51612, >IPI:IPI00014186.2|SWISS-PROT:Q15911-
1|REFSEQ_NP:NP_008816|TREMBL:Q8N2Y6|ENSEMBL:ENSP00000268489 Tax_Id=9606
Splice isoform A of Q15911 Alpha-fetoprotein enhancer binding protein

**208.** DECEG: 51624, >IPI:IPI00002657.1|SWISS-PROT:Q9UBL6-
1|REFSEQ_NP:NP_055242|ENSEMBL:ENSP00000317374 Tax_Id=9606 Splice isoform 1 of
Q9UBL6 Copine VII

**209.** DECEG: 51628, >IPI:IPI00219004.1|SWISS-PROT:Q9UBL6-
2|REFSEQ_NP:NP_705900|ENSEMBL:ENSP00000268720 Tax_Id=9606 Splice isoform 2 of
Q9UBL6 Copine VII

**210.** GEDCE: 51754, >IPI:IPI00334146.1|ENSEMBL:ENSP00000333231 Tax_Id=9606

**211.** EEGCD: 52252,
>IPI:IPI00167074.1|REFSEQ_NP:NP_689735|TREMBL:Q8N6S5|ENSEMBL:ENSP00000315357
Tax_Id=9606 Similar to putative

**212.** EGCED: 52548,
>IPI:IPI00329675.4|REFSEQ_NP:NP_777603|TREMBL:Q8N769|ENSEMBL:ENSP00000323279
Tax_Id=9606 Hypothetical protein FLJ25976

**213.** EEDGC: 52708, >IPI:IPI00246930.1|REFSEQ_XP:XP_299935 Tax_Id=9606
hypothetical protein XP_299935

**214.** GCEDE: 52936,
>IPI:IPI00007325.1|REFSEQ_NP:NP_006327|TREMBL:Q9BV66;O00156|ENSEMBL:ENSP00000
291900 Tax_Id=9606 ZYG homologue

**215.** GEECD: 53132, >IPI:IPI00220973.3|SWISS-PROT:Q9Y215-
5|REFSEQ_NP:NP_536802|ENSEMBL:ENSP00000324883 Tax_Id=9606 Splice isoform V of
Q9Y215 Acetylcholinesterase collagenic tail peptide precursor

**216.** EGEDC: 53194, >IPI:IPI00008692.1|SWISS-
PROT:O76013|REFSEQ_NP:NP_003762|TREMBL:Q86XG4|ENSEMBL:ENSP00000329272
Tax_Id=9606 Keratin, type I cuticular HA6

**217.** GEECD: 53206, >IPI:IPI00329242.1|TREMBL:Q86XK9 Tax_Id=9606 Hypothetical
protein

**218.** DGEEC: 53346,
>IPI:IPI00329260.1|REFSEQ_NP:NP_733837;NP_079414|TREMBL:Q8N3P1;Q9H9M1;Q86XN7;
Q8N3D2|ENSEMBL:ENSP00000239877;ENSP00000332034 Tax_Id=9606 KIAA2032 protein

**219.** EDGCE: 53454, >IPI:IPI00022561.1|SWISS-
PROT:O76064|REFSEQ_NP:NP_003949|ENSEMBL:ENSP00000229866 Tax_Id=9606 RING
finger protein 8

**220.** ECEDG: 53628, >IPI:IPI00008809.1|SWISS-
PROT:O76087|REFSEQ_NP:NP_001468;NP_066946|ENSEMBL:ENSP00000329716;ENSP0000033
1424;ENSP00000331056 Tax_Id=9606 GAGE-7 protein

**221.** ECEDG: 53686, >IPI:IPI00013908.1|SWISS-PROT:P25391|REFSEQ_XP:XP_209080|ENSEMBL:ENSP00000332581 Tax_Id=9606 Laminin alpha-1 chain precursor

**222.** GCDEE: 53784, >IPI:IPI00219249.2|SWISS-PROT:P78357|REFSEQ_NP:NP_003623|ENSEMBL:ENSP00000264638 Tax_Id=9606 Contactin associated protein 1 precursor

**223.** EDGEC: 54034, >IPI:IPI00247213.1|REFSEQ_XP:XP_292770 Tax_Id=9606 similar to Seminal plasma protein HSP-1

**224.** CEDGE: 54144, >IPI:IPI00247236.1|REFSEQ_XP:XP_292781 Tax_Id=9606 similar to alpha tectorin

**225.** GEECD: 54392, >IPI:IPI00013897.1|SWISS-PROT:O14672|REFSEQ_NP:NP_001101|ENSEMBL:ENSP00000260408 Tax_Id=9606 ADAM 10 precursor

**226.** CEDGE: 54624, >IPI:IPI00334240.1|ENSEMBL:ENSP00000333382 Tax_Id=9606

**227.** DCGEE: 54624, >IPI:IPI00334240.1|ENSEMBL:ENSP00000333382 Tax_Id=9606

**228.** EDCGE: 54624, >IPI:IPI00334240.1|ENSEMBL:ENSP00000333382 Tax_Id=9606

**229.** EDGEC: 54624, >IPI:IPI00334240.1|ENSEMBL:ENSP00000333382 Tax_Id=9606

**230.** GECED: 54664, >IPI:IPI00012269.1|SWISS-PROT:Q13201|REFSEQ_NP:NP_031377|ENSEMBL:ENSP00000264790 Tax_Id=9606 Endothelial cell multimerin precursor

**231.** CEDGE: 55122, >IPI:IPI00297990.1|TREMBL:Q8N7X6|ENSEMBL:ENSP00000311258 Tax_Id=9606 Hypothetical protein FLJ40237

**232.** ECGED: 55226, >IPI:IPI00028828.1|SWISS-PROT:O95125-1|REFSEQ_NP:NP_003446|TREMBL:Q9NSM4|ENSEMBL:ENSP00000299336;ENSP00000325480 Tax_Id=9606 Splice isoform Beta of O95125 Zinc finger protein 202

**233.** EEDCG: 55226, >IPI:IPI00028828.1|SWISS-PROT:O95125-1|REFSEQ_NP:NP_003446|TREMBL:Q9NSM4|ENSEMBL:ENSP00000299336;ENSP00000325480 Tax_Id=9606 Splice isoform Beta of O95125 Zinc finger protein 202

**234.** ECGED: 55228, >IPI:IPI00219137.1|SWISS-PROT:O95125-2|TREMBL:Q9Y5A5 Tax_Id=9606 Splice isoform Alpha of O95125 Zinc finger protein 202

**235.** EEDCG: 55228, >IPI:IPI00219137.1|SWISS-PROT:O95125-2|TREMBL:Q9Y5A5 Tax_Id=9606 Splice isoform Alpha of O95125 Zinc finger protein 202

**236.** EECDG: 55450, >IPI:IPI00247543.1|REFSEQ_NP:NP_055114|TREMBL:O60433|ENSEMBL:ENSP00000304499 Tax_Id=9606 R31546_1

**237.** GEECD: 55450, >IPI:IPI00247543.1|REFSEQ_NP:NP_055114|TREMBL:O60433|ENSEMBL:ENSP00000304499 Tax_Id=9606 R31546_1

**238.** EDCEG: 56090, >IPI:IPI00175989.4|REFSEQ_NP:NP_055893|TREMBL:Q8N318;O94942;Q86YR7|ENSEMBL:EN

SP00000265597;ENSP00000328118 Tax_Id=9606 Rho family guanine-nucleotide exchange factor

**239.** CEEGD: 56236, >IPI:IPI00334281.1|REFSEQ_NP:NP_861452|ENSEMBL:ENSP00000286680 Tax_Id=9606 hypothetical protein LOC286148

**240.** EEDGC: 56650, >IPI:IPI00182585.1|REFSEQ_NP:NP_003911|TREMBL:Q9UNS1;Q86VM1;Q8IWH3|ENSEMBL:ENSP00000229201 Tax_Id=9606 Timeless homolog

**241.** CDGEE: 56846, >IPI:IPI00167525.1|TREMBL:Q8N8P8 Tax_Id=9606 Hypothetical protein FLJ39054

**242.** ECEDG: 56910, >IPI:IPI00182635.2|ENSEMBL:ENSP00000319044;ENSP00000319050 Tax_Id=9606

**243.** CGDEE: 57160, >IPI:IPI00012759.1|SWISS-PROT:Q13329|REFSEQ_NP:NP_006641|ENSEMBL:ENSP00000274620 Tax_Id=9606 Complexin 2

**244.** CEDGE: 57472, >IPI:IPI00175361.2|REFSEQ_XP:XP_209869|ENSEMBL:ENSP00000333544 Tax_Id=9606 hypothetical protein XP_209869

**245.** DCEGE: 57918, >IPI:IPI00182722.1|REFSEQ_NP:NP_004839|TREMBL:O60560|ENSEMBL:ENSP00000329862 Tax_Id=9606 ICB-1

**246.** CEEGD: 58208, >IPI:IPI00298524.1|TREMBL:Q8N9E5 Tax_Id=9606 Hypothetical protein FLJ37649

**247.** DCEEG: 58828, >IPI:IPI00024254.1|SWISS-PROT:O14879|REFSEQ_NP:NP_001540|ENSEMBL:ENSP00000238972 Tax_Id=9606 Interferon-induced protein with tetratricopeptide repeats 4

**248.** DCEEG: 58842, >IPI:IPI00018300.1|SWISS-PROT:P09914|REFSEQ_NP:NP_001539|ENSEMBL:ENSP00000328042 Tax_Id=9606 Interferon-induced protein with tetratricopeptide repeats 1

**249.** GDCEE: 58904, >IPI:IPI00298624.1|TREMBL:Q9UP50 Tax_Id=9606 **Sodium** bicarbonate cotransporter

**250.** EDCEG: 58942, >IPI:IPI00001805.3|TREMBL:Q9P281|ENSEMBL:ENSP00000303486 Tax_Id=9606 Hypothetical protein KIAA1447

**251.** EEDCG: 58942, >IPI:IPI00001805.3|TREMBL:Q9P281|ENSEMBL:ENSP00000303486 Tax_Id=9606 Hypothetical protein KIAA1447

**252.** DEEGC: 58978, >IPI:IPI00054595.1|REFSEQ_NP:NP_002326|TREMBL:Q9UP66;Q96TD6;Q9UES7|ENSEMBL:ENSP00000294304 Tax_Id=9606 Low-density lipoprotein receptor-related protein 5

**253.** CEGED: 59064, >IPI:IPI00183739.1|TREMBL:Q8N9R2;Q8NAA7;Q8NCM5|ENSEMBL:ENSP00000319909 Tax_Id=9606 Hypothetical protein FLJ36696

**254.** CEEGD: 59148, >IPI:IPI00334393.1|REFSEQ_XP:XP_209917 Tax_Id=9606 similar to hypothetical protein FLJ32949

**255.** GDECE: 59304, >IPI:IPI00167814.1|TREMBL:Q8N9U3 Tax_Id=9606 Hypothetical protein FLJ36512

**256.** GEECD: 59348, >IPI:IPI00021905.1|SWISS-PROT:Q13443|REFSEQ_NP:NP_003807|TREMBL:Q8NFM6|ENSEMBL:ENSP00000305538 Tax_Id=9606 ADAM 9 precursor

**257.** EGCDE: 59580, >IPI:IPI00298758.1|SWISS-PROT:O60602|REFSEQ_NP:NP_003259 Tax_Id=9606 Toll-like receptor 5 precursor

**258.** GCDEE: 59580, >IPI:IPI00298758.1|SWISS-PROT:O60602|REFSEQ_NP:NP_003259 Tax_Id=9606 Toll-like receptor 5 precursor

**259.** ECDEG: 59660, >IPI:IPI00018370.1|REFSEQ_NP:NP_003165|TREMBL:O60612|ENSEMBL:ENSP00000263058 Tax_Id=9606 Supervillin

**260.** CDGEE: 60296, >IPI:IPI00020929.1|REFSEQ_NP:NP_150643|TREMBL:O14968|ENSEMBL:ENSP00000321568 Tax_Id=9606 ApoER2delta4-7

**261.** ECEDG: 60306, >IPI:IPI00024273.1|SWISS-PROT:P98155-1|REFSEQ_NP:NP_003374|ENSEMBL:ENSP00000327160 Tax_Id=9606 Splice isoform Long of P98155 Very low-density lipoprotein receptor precursor

**262.** ECEDG: 60308, >IPI:IPI00219353.1|SWISS-PROT:P98155-2 Tax_Id=9606 Splice isoform Short of P98155 Very low-density lipoprotein receptor precursor

**263.** EEGDC: 60386, >IPI:IPI00004358.2|SWISS-PROT:P11216|REFSEQ_NP:NP_002853|TREMBL:Q8TDG6|ENSEMBL:ENSP00000216962 Tax_Id=9606 Glycogen phosphorylase, brain form

**264.** EEGDC: 60398, >IPI:IPI00023177.1|SWISS-PROT:Q13111-1|REFSEQ_NP:NP_005474|ENSEMBL:ENSP00000301280 Tax_Id=9606 Splice isoform 1 of Q13111 Chromatin assembly factor 1 subunit A

**265.** EDEGC: 60868, >IPI:IPI00179185.1|REFSEQ_NP:NP_003643|TREMBL:O00520;Q9BRA7|ENSEMBL:ENSP00000315074 Tax_Id=9606 Carboxypeptidase Z precursor

**266.** ECEGD: 60924, >IPI:IPI00248855.1|REFSEQ_XP:XP_294313 Tax_Id=9606 similar to Multifunctional protein ADE2

**267.** GEEDC: 61238, >IPI:IPI00028136.1|REFSEQ_NP:NP_066978|TREMBL:Q96QQ4;Q96QQ0;Q9NNX6;Q96QQ8|ENSEMBL:ENSP00000315477 Tax_Id=9606 Membrane-associated lectin type-C

**268.** ECGED: 61396, >IPI:IPI00000151.1|SWISS-PROT:P18564|REFSEQ_NP:NP_000879|ENSEMBL:ENSP00000283249 Tax_Id=9606 Integrin beta-6 precursor

**269.** ECEDG: 61406, >IPI:IPI00010746.1|SWISS-PROT:P48651|REFSEQ_NP:NP_055569|TREMBL:Q9BSY0|ENSEMBL:ENSP00000287028 Tax_Id=9606 Phosphatidylserine synthase I

**270.** GEEDC: 61658, >IPI:IPI00334503.1|REFSEQ_NP:NP_065175|TREMBL:Q9UFH7;Q96GN2;Q9H6B7|ENSEMBL:ENSP00000319338 Tax_Id=9606 KIAA1885 protein

**271.** ECDEG: 61774, >IPI:IPI00014310.1|SWISS-PROT:Q13616|REFSEQ_NP:NP_003583|ENSEMBL:ENSP00000326804 Tax_Id=9606 Cullin homolog 1

**272.** CEDGE: 61992, >IPI:IPI00299219.1|SWISS-PROT:O00622|REFSEQ_NP:NP_001545|ENSEMBL:ENSP00000271019 Tax_Id=9606 CYR61 protein precursor

**273.** CEDEG: 62044, >IPI:IPI00007365.1|SWISS-PROT:O00635|REFSEQ_NP:NP_006346|ENSEMBL:ENSP00000230099 Tax_Id=9606 RING finger protein 15

**274.** CDGEE: 62288, >IPI:IPI00249276.1|REFSEQ_XP:XP_294475 Tax_Id=9606 similar to Asparaginyl-tRNA synthetase, cytoplasmic (Asparagine--tRNA ligase) (AsnRS)

**275.** CDGEE: 62934, >IPI:IPI00012793.1|SWISS-PROT:Q13342-1|REFSEQ_NP:NP_009168|TREMBL:Q8IWJ1 Tax_Id=9606 Splice isoform LYSp100-B of Q13342 LYSp100 protein

**276.** CDGEE: 62938, >IPI:IPI00219535.1|SWISS-PROT:Q13342-2 Tax_Id=9606 Splice isoform LYSp100-A of Q13342 LYSp100 protein

**277.** CDGEE: 62940, >IPI:IPI00219536.1|SWISS-PROT:Q13342-3|ENSEMBL:ENSP00000181092 Tax_Id=9606 Splice isoform Sp140 of Q13342 LYSp100 protein

**278.** DCGEE: 62964, >IPI:IPI00014972.1|TREMBL:Q9NPK8|ENSEMBL:ENSP00000309032;ENSP00000328558 Tax_Id=9606 DJ34F7.1.2 (Tenascin XB

**279.** EDCGE: 62964, >IPI:IPI00014972.1|TREMBL:Q9NPK8|ENSEMBL:ENSP00000309032;ENSP00000328558 Tax_Id=9606 DJ34F7.1.2 (Tenascin XB

**280.** CEDGE: 62966, >IPI:IPI00334575.1|TREMBL:Q9NPK9|ENSEMBL:ENSP00000330143 Tax_Id=9606 DJ34F7.1.1 (Tenascin XB

**281.** DCGEE: 62966, >IPI:IPI00334575.1|TREMBL:Q9NPK9|ENSEMBL:ENSP00000330143 Tax_Id=9606 DJ34F7.1.1 (Tenascin XB

**282.** EDCGE: 62966, >IPI:IPI00334575.1|TREMBL:Q9NPK9|ENSEMBL:ENSP00000330143 Tax_Id=9606 DJ34F7.1.1 (Tenascin XB

**283.** EDGEC: 62966, >IPI:IPI00334575.1|TREMBL:Q9NPK9|ENSEMBL:ENSP00000330143 Tax_Id=9606 DJ34F7.1.1 (Tenascin XB

**284.** EEGCD: 63136, >IPI:IPI00023014.1|SWISS-PROT:P04275|REFSEQ_NP:NP_000543|TREMBL:Q99806;Q9UD22|ENSEMBL:ENSP00000261405 Tax_Id=9606 Von Willebrand factor precursor

**285.** ECGDE: 63610, >IPI:IPI00033553.1|REFSEQ_NP:NP_065106|TREMBL:Q9H847;Q9NQ90|ENSEMBL:ENSP00000314048 Tax_Id=9606 Hypothetical protein

**286.** DCGEE: 63848, >IPI:IPI00011901.2|SWISS-PROT:Q9H013-1|REFSEQ_NP:NP_075525|TREMBL:Q8TBU7;Q8N504 Tax_Id=9606 Splice isoform A of Q9H013 ADAM 19 precursor

**287.** DGEEC: 63848, >IPI:IPI00011901.2|SWISS-PROT:Q9H013-1|REFSEQ_NP:NP_075525|TREMBL:Q8TBU7;Q8N504 Tax_Id=9606 Splice isoform A of Q9H013 ADAM 19 precursor

**288.** GEECD: 63848, >IPI:IPI00011901.2|SWISS-PROT:Q9H013-1|REFSEQ_NP:NP_075525|TREMBL:Q8TBU7;Q8N504 Tax_Id=9606 Splice isoform A of Q9H013 ADAM 19 precursor

**289.** DCGEE: 63850, >IPI:IPI00249735.1|SWISS-PROT:Q9H013-2|REFSEQ_NP:NP_150377|ENSEMBL:ENSP00000257527 Tax_Id=9606 Splice isoform B of Q9H013 ADAM 19 precursor

**290.** DGEEC: 63850, >IPI:IPI00249735.1|SWISS-PROT:Q9H013-2|REFSEQ_NP:NP_150377|ENSEMBL:ENSP00000257527 Tax_Id=9606 Splice isoform B of Q9H013 ADAM 19 precursor

**291.** GEECD: 63850, >IPI:IPI00249735.1|SWISS-PROT:Q9H013-2|REFSEQ_NP:NP_150377|ENSEMBL:ENSP00000257527 Tax_Id=9606 Splice isoform B of Q9H013 ADAM 19 precursor

**292.** GEECD: 64386, >IPI:IPI00299652.1|SWISS-PROT:O75078-1|REFSEQ_NP:NP_002381|ENSEMBL:ENSP00000200557 Tax_Id=9606 Splice isoform Long of O75078 ADAM 11 precursor

**293.** GEECD: 64388, >IPI:IPI00299653.1|SWISS-PROT:O75078-2|REFSEQ_NP:NP_067625 Tax_Id=9606 Splice isoform Short of O75078 ADAM 11 precursor

**294.** CEEDG: 64522, >IPI:IPI00021427.1|SWISS-PROT:Q9NZL6-1 Tax_Id=9606 Splice isoform A of Q9NZL6 Ral guanine nucleotide dissociation stimulator-like 1

**295.** CEEDG: 64528, >IPI:IPI00299679.1|SWISS-PROT:Q9NZL6-2|REFSEQ_NP:NP_055964|ENSEMBL:ENSP00000303192 Tax_Id=9606 Splice isoform B of Q9NZL6 Ral guanine nucleotide dissociation stimulator-like 1

**296.** CEDGE: 64816, >IPI:IPI00334636.1|ENSEMBL:ENSP00000328067 Tax_Id=9606

**297.** DCGEE: 64816, >IPI:IPI00334636.1|ENSEMBL:ENSP00000328067 Tax_Id=9606

**298.** EDGEC: 64816, >IPI:IPI00334636.1|ENSEMBL:ENSP00000328067 Tax_Id=9606

**299.** EGDEC: 65012, >IPI:IPI00033970.1|SWISS-PROT:Q9H2R5-1|REFSEQ_NP:NP_059979|ENSEMBL:ENSP00000301421 Tax_Id=9606 Splice isoform 1 of Q9H2R5 Kallikrein 15 precursor

**300.** EGDEC: 65014, >IPI:IPI00150552.1|SWISS-PROT:Q9H2R5-2|REFSEQ_NP:NP_075382 Tax_Id=9606 Splice isoform 2 of Q9H2R5 Kallikrein 15 precursor

**301.** EGDEC: 65018, >IPI:IPI00219692.1|SWISS-PROT:Q9H2R5-3|REFSEQ_NP:NP_612630|TREMBL:Q96RQ0 Tax_Id=9606 Splice isoform 3 of Q9H2R5 Kallikrein 15 precursor

**302.** EGDEC: 65020, >IPI:IPI00219693.1|SWISS-PROT:Q9H2R5-4|REFSEQ_NP:NP_612631|ENSEMBL:ENSP00000314783 Tax_Id=9606 Splice isoform 4 of Q9H2R5 Kallikrein 15 precursor

**303.** GEECD: 65516, >IPI:IPI00018146.1|SWISS-PROT:P27348|REFSEQ_NP:NP_006817|TREMBL:Q9UP48|ENSEMBL:ENSP00000238081 Tax_Id=9606 14-3-3 protein tau

**304.** GDCEE: 66052, >IPI:IPI00016949.1|TREMBL:Q9NRZ1|ENSEMBL:ENSP00000307349 Tax_Id=9606 **Sodium** bicarbonate cotransporter NBC1

**305.** EDGCE: 66446, >IPI:IPI00021466.1|REFSEQ_NP:NP_006442|TREMBL:Q9H074|ENSEMBL:ENSP00000302768 Tax_Id=9606 Hypothetical protein

**306.** CEGED: 66934, >IPI:IPI00337657.1|REFSEQ_NP:NP_694972|TREMBL:Q96NK2|ENSEMBL:ENSP00000318348 Tax_Id=9606 nephrocystin 3

**307.** EEDGC: 66976, >IPI:IPI00250876.1|REFSEQ_XP:XP_296098 Tax_Id=9606 hypothetical protein XP_296098

**308.** CEGED: 67040, >IPI:IPI00100787.1|REFSEQ_NP:NP_115636|TREMBL:Q9H0B2|ENSEMBL:ENSP00000272454;ENSP00000330023;ENSP00000327486 Tax_Id=9606 Hypothetical protein

**309.** DCEGE: 67040, >IPI:IPI00100787.1|REFSEQ_NP:NP_115636|TREMBL:Q9H0B2|ENSEMBL:ENSP00000272454;ENSP00000330023;ENSP00000327486 Tax_Id=9606 Hypothetical protein

**310.** EECGD: 67796, >IPI:IPI00006171.2|REFSEQ_NP:NP_006156|TREMBL:Q12869;Q15312;Q9H048|ENSEMBL:ENSP00000303800;ENSP00000315172 Tax_Id=9606 R kappa B

**311.** ECEDG: 68094, >IPI:IPI00016645.1|SWISS-PROT:Q15375|REFSEQ_NP:NP_004431|TREMBL:Q8N368|ENSEMBL:ENSP00000257785 Tax_Id=9606 Ephrin type-A receptor 7 precursor

**312.** EDEGC: 69284, >IPI:IPI00300576.1|SWISS-PROT:P43220|REFSEQ_NP:NP_002053|ENSEMBL:ENSP00000229900 Tax_Id=9606 Glucagon-like peptide 1 receptor precursor

**313.** CEDGE: 69404, >IPI:IPI00334817.1|ENSEMBL:ENSP00000328379 Tax_Id=9606

**314.** ECEDG: 69906, >IPI:IPI00168052.1|TREMBL:Q8NAN7 Tax_Id=9606 Hypothetical protein FLJ35062

**315.** ECDGE: 70266, >IPI:IPI00057451.1|REFSEQ_XP:XP_060355 Tax_Id=9606 similar to RIKEN cDNA 1810030J14

**316.** EDECG: 70422, >IPI:IPI00329580.4|REFSEQ_NP:NP_689966|TREMBL:Q8IX30;Q8NAV8;Q8NAU9|ENSEMBL:ENSP00000274938 Tax_Id=9606 Hypothetical protein FLJ34743

**317.** GEECD: 71768, >IPI:IPI00023134.1|SWISS-PROT:Q9H2U9|REFSEQ_NP:NP_003808 Tax_Id=9606 ADAM 7 precursor

**318.** CEDGE: 71836, >IPI:IPI00170865.1|REFSEQ_NP:NP_690864|TREMBL:Q9H2V6|ENSEMBL:ENSP00000261449 Tax_Id=9606 Membrane-associated guanylate kinase-related MAGI-3

**319.** DEEGC: 72098, >IPI:IPI00057797.4|REFSEQ_XP:XP_060405 Tax_Id=9606 similar to MEGF6

**320.** CEDGE: 72452, >IPI:IPI00252375.1|REFSEQ_XP:XP_296374 Tax_Id=9606 hypothetical protein XP_296374

**321.** EECDG: 73116, >IPI:IPI00146811.1|REFSEQ_XP:XP_301149 Tax_Id=9606 similar to hypothetical protein

**322.** ECEDG: 73550, >IPI:IPI00334972.1|ENSEMBL:ENSP00000328574;ENSP00000332664 Tax_Id=9606

**323.** CEGDE: 74738, >IPI:IPI00016724.1|REFSEQ_NP:NP_060393|TREMBL:Q8TCQ1;Q9NWR0|ENSEMBL:ENSP00000274056 Tax_Id=9606 Hypothetical protein FLJ20668

**324.** DCGEE: 74744, >IPI:IPI00069363.1|REFSEQ_XP:XP_016093|ENSEMBL:ENSP00000316037 Tax_Id=9606 similar to eukaryotic initiation factor 5A

**325.** ECGED: 74890, >IPI:IPI00301812.1|REFSEQ_NP:NP_071420|TREMBL:Q9H4F8;Q96F78|ENSEMBL:ENSP00000238629 Tax_Id=9606 Secreted modular **calcium**-binding protein precursor

**326.** EEDGC: 75218, >IPI:IPI00253046.1|REFSEQ_XP:XP_301273|ENSEMBL:ENSP00000303287 Tax_Id=9606 similar to hypothetical protein

**327.** GECDE: 75998, >IPI:IPI00253274.1|REFSEQ_XP:XP_296525 Tax_Id=9606 hypothetical protein XP_296525

**328.** EDECG: 76060, >IPI:IPI00253294.1|REFSEQ_XP:XP_296538 Tax_Id=9606 hypothetical protein XP_296538

**329.** EGCED: 76404, >IPI:IPI00176387.1|TREMBL:Q8NE58|ENSEMBL:ENSP00000313062 Tax_Id=9606 Hypothetical protein

**330.** ECGED: 76536, >IPI:IPI00168623.1|TREMBL:Q8NE87|ENSEMBL:ENSP00000324097 Tax_Id=9606 Hypothetical protein

**331.** DCEGE: 76560, >IPI:IPI00014890.1|SWISS-PROT:Q9NXR5|REFSEQ_NP:NP_060134|TREMBL:Q9NXQ9;Q9H6D6;Q8IUW1|ENSEMBL:ENSP00000267339 Tax_Id=9606 Ankyrin repeat domain protein 10

**332.** EGDCE: 77184, >IPI:IPI00025365.1|SWISS-PROT:P14138-1|REFSEQ_NP:NP_000105|ENSEMBL:ENSP00000311854;ENSP00000326965 Tax_Id=9606 Splice isoform Long of P14138 Endothelin-3 precursor

**333.** GDCEE: 77184, >IPI:IPI00025365.1|SWISS-PROT:P14138-1|REFSEQ_NP:NP_000105|ENSEMBL:ENSP00000311854;ENSP00000326965 Tax_Id=9606 Splice isoform Long of P14138 Endothelin-3 precursor

**334.** EGDCE: 77186, >IPI:IPI00220210.1|SWISS-PROT:P14138-2 Tax_Id=9606 Splice isoform Short of P14138 Endothelin-3 precursor

**335.** GDCEE: 77186, >IPI:IPI00220210.1|SWISS-PROT:P14138-2 Tax_Id=9606 Splice isoform Short of P14138 Endothelin-3 precursor

**336.** DEGCE: 77234, >IPI:IPI00253545.1|REFSEQ_XP:XP_301395 Tax_Id=9606 similar to neuronal apoptosis inhibitor protein 9

**337.** EGCED: 77234, >IPI:IPI00253545.1|REFSEQ_XP:XP_301395 Tax_Id=9606 similar to neuronal apoptosis inhibitor protein 9

**338.** CEDGE: 77406, >IPI:IPI00302351.1|TREMBL:Q8NEF5 Tax_Id=9606 Similar to serine/threonine kinase 33

**339.** GDCEE: 77704, >IPI:IPI00168680.1|TREMBL:Q8NEJ2 Tax_Id=9606 Similar to solute carrier family 4, **sodium** bicarbonate cotransporter, member 4

**340.** CDGEE: 78456, >IPI:IPI00220259.1|SWISS-PROT:O95613|REFSEQ_NP:NP_006022|TREMBL:Q8IWJ7|ENSEMBL:ENSP00000291695 Tax_Id=9606 pericentrin B

**341.** GEDCE: 78564, >IPI:IPI00015345.1|SWISS-PROT:Q9NYQ7|REFSEQ_NP:NP_001398|ENSEMBL:ENSP00000164024 Tax_Id=9606 Cadherin EGF LAG seven-pass G-type receptor 3 precursor

**342.** DCGEE: 79218, >IPI:IPI00165045.1|TREMBL:Q9NYZ5|ENSEMBL:ENSP00000327220 Tax_Id=9606 **Calcium** channel alpha1E subunit, delta19 delta40 delta46 splice variant

**343.** EDEGC: 80064, >IPI:IPI00335249.1|TREMBL:Q9H6Y0|ENSEMBL:ENSP00000329394 Tax_Id=9606 Hypothetical protein FLJ21709

**344.** EGCDE: 80226, >IPI:IPI00254385.1|REFSEQ_XP:XP_296731 Tax_Id=9606 hypothetical protein XP_296731

**345.** EGDCE: 80386, >IPI:IPI00002849.1|SWISS-PROT:P28324-1|REFSEQ_NP:NP_001964|TREMBL:Q8IXL1|ENSEMBL:ENSP00000289703 Tax_Id=9606 Splice isoform 1 of P28324 ETS-domain protein ELK-4

**346.** EGDCE: 80392, >IPI:IPI00220318.1|SWISS-PROT:P28324-2|REFSEQ_NP:NP_068567 Tax_Id=9606 Splice isoform 2 of P28324 ETS-domain protein ELK-4

**347.** EECGD: 80622, >IPI:IPI00025803.2|SWISS-PROT:P06213-1|REFSEQ_NP:NP_000199|TREMBL:Q86WY9;Q9UCB7;Q9UCB8;Q9UCB9|ENSEMBL:ENSP00000303830 Tax_Id=9606 Splice isoform Long of P06213 Insulin receptor precursor

**348.** EECGD: 80624, >IPI:IPI00220325.2|SWISS-PROT:P06213-2 Tax_Id=9606 Splice isoform Short of P06213 Insulin receptor precursor

**349.** CGDEE: 80916, >IPI:IPI00032063.1|REFSEQ_NP:NP_061027|TREMBL:Q8WY28;Q8WY27;Q9NZR2;Q8WY30;Q8WY31;Q8WY26;Q8WY29|ENSEMBL:ENSP00000307314;ENSP00000316011;ENSP00000333057 Tax_Id=9606 Low density lipoprotein receptor related protein-deleted in tumor

**350.** EGEDC: 80918, >IPI:IPI00157417.1|SWISS-PROT:Q9BYH1-1|REFSEQ_NP:NP_066938|ENSEMBL:ENSP00000215905 Tax_Id=9606 Splice isoform 4 of Q9BYH1 Seizure 6-like protein precursor

**351.** EGEDC: 80926, >IPI:IPI00163788.5|SWISS-PROT:Q9BYH1-2|ENSEMBL:ENSP00000248933 Tax_Id=9606 Splice isoform 1 of Q9BYH1 Seizure 6-like protein precursor

**352.** EGEDC: 80928, >IPI:IPI00220333.1|SWISS-PROT:Q9BYH1-3 Tax_Id=9606 Splice isoform 2 of Q9BYH1 Seizure 6-like protein precursor

**353.** EGEDC: 80932, >IPI:IPI00220334.1|SWISS-PROT:Q9BYH1-4 Tax_Id=9606 Splice isoform 3 of Q9BYH1 Seizure 6-like protein precursor

**354.** EDGCE: 81198, >IPI:IPI00303274.1|TREMBL:Q96B61 Tax_Id=9606 Polyadenylate binding protein-interacting protein 1

**355.** DCGEE: 81792, >IPI:IPI00335323.1|ENSEMBL:ENSP00000330151 Tax_Id=9606

**356.** DGECE: 81834, >IPI:IPI00023824.1|SWISS-PROT:P98095|REFSEQ_NP:NP_001989|TREMBL:Q86V58|ENSEMBL:ENSP00000295760 Tax_Id=9606 Fibulin-2 precursor

**357.** GECED: 81834, >IPI:IPI00023824.1|SWISS-PROT:P98095|REFSEQ_NP:NP_001989|TREMBL:Q86V58|ENSEMBL:ENSP00000295760 Tax_Id=9606 Fibulin-2 precursor

**358.** ECGDE: 82610, >IPI:IPI00328847.2|REFSEQ_NP:NP_079047|TREMBL:Q9H897;Q86UY6|ENSEMBL:ENSP00000265463 Tax_Id=9606 Hypothetical protein FLJ13848

**359.** GEDCE: 82722, >IPI:IPI00303595.1|TREMBL:Q96BJ0|ENSEMBL:ENSP00000253015 Tax_Id=9606 Similar to uridine-cytidine kinase 1

**360.** EGCED: 83570, >IPI:IPI00100933.1|SWISS-PROT:Q96BW5|REFSEQ_NP:NP_109589|ENSEMBL:ENSP00000298942 Tax_Id=9606 Phosphotriesterase related protein

**361.** EDGCE: 83906, >IPI:IPI00024292.1|SWISS-PROT:P98164|REFSEQ_NP:NP_004516|TREMBL:Q9NP34|ENSEMBL:ENSP00000263816 Tax_Id=9606 Low-density lipoprotein receptor-related protein 2 precursor

**362.** ECEDG: 84438, >IPI:IPI00303963.1|SWISS-PROT:P06681|REFSEQ_NP:NP_000054|TREMBL:Q95IG1;O19694;Q8N6L6;Q86SV5|ENSEMBL:ENSP00000299367;ENSP00000319023;ENSP00000330581 Tax_Id=9606 Complement C2 precursor

**363.** CDEGE: 84848, >IPI:IPI00169309.1|REFSEQ_NP:NP_689566|TREMBL:Q8NHS1|ENSEMBL:ENSP00000291715 Tax_Id=9606 Similar to RIKEN cDNA 1700071E18 gene

**364.** ECEGD: 84966, >IPI:IPI00255906.1|REFSEQ_XP:XP_296987 Tax_Id=9606 hypothetical protein XP_296987

**365.** EDEGC: 85920, >IPI:IPI00335481.1|ENSEMBL:ENSP00000226892 Tax_Id=9606

**366.** CDEEG: 86376, >IPI:IPI00337759.1|REFSEQ_NP:NP_689961|TREMBL:Q96DP2|ENSEMBL:ENSP00000297222 Tax_Id=9606 hypothetical protein FLJ31340

**367.** ECEGD: 87222, >IPI:IPI00304540.4|TREMBL:Q9C0A2;Q8WUN4;Q8N598;Q96SN2|REFSEQ_XP:XP_042708|ENSEMBL:ENSP00000322449 Tax_Id=9606 Hypothetical protein KIAA1761

**368.** EGDEC: 87286, >IPI:IPI00028276.1|TREMBL:Q9C0B0|REFSEQ_XP:XP_036115|ENSEMBL:ENSP00000293218 Tax_Id=9606 Hypothetical protein KIAA1753

**369.** EEDGC: 88108, >IPI:IPI00256910.1|REFSEQ_XP:XP_301892 Tax_Id=9606 hypothetical protein XP_301892

**370.** EEDGC: 88170, >IPI:IPI00335541.1|TREMBL:O94802 Tax_Id=9606 HTIM1

**371.** GEDEC: 88330, >IPI:IPI00006056.1|SWISS-PROT:P36915|REFSEQ_NP:NP_005266|TREMBL:Q96CT5;Q96QB8|ENSEMBL:ENSP00000259886;ENSP00000319707;ENSP00000316778 Tax_Id=9606 Guanine nucleotide-binding protein-like 1

**372.** GEEDC: 88688, >IPI:IPI00335574.1|ENSEMBL:ENSP00000316092 Tax_Id=9606

**373.** DCGEE: 88806, >IPI:IPI00257026.1|REFSEQ_XP:XP_303108 Tax_Id=9606 hypothetical protein XP_303108

**374.** GEECD: 89054, >IPI:IPI00000779.1|SWISS-PROT:Q9P0K1-1|REFSEQ_NP:NP_068369|TREMBL:Q8IYE7;Q9UKK0;Q86UM2|ENSEMBL:ENSP00000265727 Tax_Id=9606 Splice isoform 1 of Q9P0K1 ADAM 22 precursor

**375.** GEECD: 89060, >IPI:IPI00220631.1|SWISS-PROT:Q9P0K1-2|REFSEQ_NP:NP_068368|ENSEMBL:ENSP00000315900 Tax_Id=9606 Splice isoform 2 of Q9P0K1 ADAM 22 precursor

**376.** GEECD: 89068, >IPI:IPI00220632.1|SWISS-PROT:Q9P0K1-3|REFSEQ_NP:NP_004185 Tax_Id=9606 Splice isoform 3 of Q9P0K1 ADAM 22 precursor

**377.** GEECD: 89076, >IPI:IPI00220634.1|SWISS-PROT:Q9P0K1-4|REFSEQ_NP:NP_068367 Tax_Id=9606 Splice isoform 4 of Q9P0K1 ADAM 22 precursor

**378.** GEECD: 89080, >IPI:IPI00220635.1|SWISS-PROT:Q9P0K1-5|REFSEQ_NP:NP_057435 Tax_Id=9606 Splice isoform 5 of Q9P0K1 ADAM 22 precursor

**379.** EDGEC: 89742, >IPI:IPI00027473.1|SWISS-PROT:P08172|REFSEQ_NP:NP_000730|TREMBL:Q86SJ1;Q96RH0|ENSEMBL:ENSP00000319984 Tax_Id=9606 Muscarinic acetylcholine receptor M2

**380.** ECEDG: 89816, >IPI:IPI00335591.1|ENSEMBL:ENSP00000330504;ENSP00000332566 Tax_Id=9606

**381.** DEECG: 90054, >IPI:IPI00016353.1|SWISS-PROT:O94907|REFSEQ_NP:NP_036374|ENSEMBL:ENSP00000224974 Tax_Id=9606 Dickkopf related protein-1 precursor

**382.** EDGCE: 90668, >IPI:IPI00337775.1|REFSEQ_NP:NP_877590|TREMBL:Q9BS63 Tax_Id=9606 Similar to polyadenylate binding protein-interacting protein 1

**383.** GEECD: 91036, >IPI:IPI00337776.1|REFSEQ_XP:XP_291251|ENSEMBL:ENSP00000175238 Tax_Id=9606 similar to ADAM 7 precursor (A disintegrin and metalloproteinase domain 7) (Sperm maturation-related glycoprotein GP-83)

**384.** CEGED: 91690, >IPI:IPI00305390.1|SWISS-PROT:P52789 Tax_Id=9606 Hexokinase, type II

**385.** ECGDE: 91888, >IPI:IPI00257749.1|REFSEQ_XP:XP_298505 Tax_Id=9606 hypothetical protein XP_298505

**386.** CEGED: 92126, >IPI:IPI00182473.2|REFSEQ_XP:XP_208296|ENSEMBL:ENSP00000283154;ENSP00000303659 Tax_Id=9606 similar to RAN-binding protein 2-like 1 isoform 1

**387.** DCEGE: 92126, >IPI:IPI00182473.2|REFSEQ_XP:XP_208296|ENSEMBL:ENSP00000283154;ENSP00000303659 Tax_Id=9606 similar to RAN-binding protein 2-like 1 isoform 1

**388.** EGEDC: 92248, >IPI:IPI00257843.1|REFSEQ_XP:XP_298576 Tax_Id=9606 hypothetical protein XP_298576

**389.** CEEGD: 92738, >IPI:IPI00186138.1|ENSEMBL:ENSP00000295809 Tax_Id=9606

**390.** GCEED: 92890, >IPI:IPI00021701.1|REFSEQ_NP:NP_056031|TREMBL:O43314|ENSEMBL:ENSP00000274387 Tax_Id=9606 Hypothetical protein KIAA0433

**391.** ECDEG: 92984, >IPI:IPI00258019.1|REFSEQ_XP:XP_291378|ENSEMBL:ENSP00000289853 Tax_Id=9606 similar to C4b-binding protein alpha chain precursor (C4bp) (Proline-rich protein) (PRP)

**392.** ECGED: 93020, >IPI:IPI00305608.1|TREMBL:O43339|REFSEQ_XP:XP_209111|ENSEMBL:ENSP00000303820 Tax_Id=9606 R28830_1

**393.** ECEGD: 93236, >IPI:IPI00335713.1|REFSEQ_NP:NP_006759|TREMBL:O43238|ENSEMBL:ENSP00000330813 Tax_Id=9606 BRCA1 associated protein

**394.** CEGDE: 94552, >IPI:IPI00063121.1|REFSEQ_NP:NP_542381|TREMBL:Q96HJ6|ENSEMBL:ENSP00000256538 Tax_Id=9606 Similar to RIKEN cDNA 5730421E18 gene

**395.** DGCEE: 94990, >IPI:IPI00106955.1|TREMBL:Q9BUA3 Tax_Id=9606 Hypothetical protein

**396.** CGDEE: 95136, >IPI:IPI00063273.1|SWISS-PROT:Q96I34|REFSEQ_NP:NP_116291|ENSEMBL:ENSP00000292539 Tax_Id=9606 Protein phosphatase 1 regulatory inhibitor subunit 16A

**397.** DCEGE: 95884, >IPI:IPI00004437.1|TREMBL:Q9BV12|ENSEMBL:ENSP00000312534 Tax_Id=9606 Similar to hypothetical protein FLJ20093

**398.** CGEED: 96084, >IPI:IPI00063592.1|REFSEQ_NP:NP_543137|TREMBL:Q96RY2;Q8WXK5;Q96IE6;Q86X18|ENSEMBL:ENSP00000303779 Tax_Id=9606 Similar to RIKEN cDNA 2310012N15 gene

**399.** DEEGC: 96108, >IPI:IPI00063605.1|REFSEQ_NP:NP_116265|TREMBL:Q96IF1|ENSEMBL:ENSP00000262713;ENSP00000331606 Tax_Id=9606 Similar to ajuba

**400.** EGCED: 96108, >IPI:IPI00063605.1|REFSEQ_NP:NP_116265|TREMBL:Q96IF1|ENSEMBL:ENSP00000262713;ENSP00000331606 Tax_Id=9606 Similar to ajuba

**401.** DGCEE: 96196, >IPI:IPI00063633.1|TREMBL:Q96IH0|REFSEQ_XP:XP_290508|ENSEMBL:ENSP00000294244 Tax_Id=9606 Hypothetical protein

**402.** GEECD: 96372, >IPI:IPI00031687.1|SWISS-PROT:Q9Y215-1|REFSEQ_NP:NP_005668 Tax_Id=9606 Splice isoform I of Q9Y215 Acetylcholinesterase collagenic tail peptide precursor

**403.** GEECD: 96374, >IPI:IPI00220970.1|SWISS-PROT:Q9Y215-2|REFSEQ_NP:NP_536799|ENSEMBL:ENSP00000326594 Tax_Id=9606 Splice isoform II of Q9Y215 Acetylcholinesterase collagenic tail peptide precursor

**404.** GEECD: 96378, >IPI:IPI00220971.1|SWISS-PROT:Q9Y215-3 Tax_Id=9606 Splice isoform III of Q9Y215 Acetylcholinesterase collagenic tail peptide precursor

**405.** GEECD: 96386, >IPI:IPI00220972.1|SWISS-PROT:Q9Y215-4|REFSEQ_NP:NP_536801 Tax_Id=9606 Splice isoform IV of Q9Y215 Acetylcholinesterase collagenic tail peptide precursor

**406.** GDCEE: 96466, >IPI:IPI00258909.1|REFSEQ_XP:XP_303547 Tax_Id=9606 hypothetical protein XP_303547

**407.** GEECD: 96498, >IPI:IPI00306205.1|SWISS-PROT:O43506|REFSEQ_NP:NP_003805|ENSEMBL:ENSP00000256389 Tax_Id=9606 ADAM 20 precursor

**408.** ECDGE: 96822, >IPI:IPI00335864.1|ENSEMBL:ENSP00000332147 Tax_Id=9606

**409.** CDGEE: 97348, >IPI:IPI00294137.3|REFSEQ_NP:NP_059992|TREMBL:Q99876|ENSEMBL:ENSP00000303634 Tax_Id=9606 apolipoprotein E receptor 2 isoform 3 precursor

**410.** GEECD: 97714, >IPI:IPI00259271.1|REFSEQ_XP:XP_298879 Tax_Id=9606 hypothetical protein XP_298879

**411.** DECGE: 98628, >IPI:IPI00064242.1|TREMBL:Q96JP8 Tax_Id=9606 Fibrillin3

**412.** EDECG: 98628, >IPI:IPI00064242.1|TREMBL:Q96JP8 Tax_Id=9606 Fibrillin3

**413.** ECGED: 98744, >IPI:IPI00335965.1|ENSEMBL:ENSP00000332244 Tax_Id=9606

**414.** GEDCE: 99692, >IPI:IPI00027771.1|SWISS-PROT:Q9HA47|REFSEQ_NP:NP_113620|ENSEMBL:ENSP00000298508 Tax_Id=9606 Uridine-cytidine kinase 1

**415.** GEECD: 100172, >IPI:IPI00021903.1|REFSEQ_NP:NP_003803|TREMBL:O75077;Q9UKK1|ENSEMBL:ENSP00000264377 Tax_Id=9606 MDC3

**416.** GEDCE: 100292, >IPI:IPI00306850.1|TREMBL:O75095|REFSEQ_XP:XP_031401|ENSEMBL:ENSP00000294599 Tax_Id=9606 MEGF6

**417.** GEECD: 101716, >IPI:IPI00013569.1|REFSEQ_NP:NP_064714|TREMBL:Q9BXP8;Q96PH8;Q9H4C9|ENSEMBL:ENSP00000263529 Tax_Id=9606 Pregnancy-associated plasma preproprotein-A2

**418.** CGEDE: 102056, >IPI:IPI00307150.1|REFSEQ_NP:NP_065801|TREMBL:Q96HN3;Q9HAV4;Q9ULC9;Q9BWM6;Q9H9M4;Q9NT89;Q9NW39;Q96G48;Q9H8N6;Q9BZV5|ENSEMBL:ENSP00000265351 Tax_Id=9606 Exportin 5

**419.** CEDGE: 102110, >IPI:IPI00012472.1|SWISS-PROT:P31645|REFSEQ_NP:NP_001036|TREMBL:Q9NYN7|ENSEMBL:ENSP00000261707 Tax_Id=9606 **Sodium**-dependent serotonin transporter

**420.** EECGD: 102130, >IPI:IPI00260357.1|REFSEQ_XP:XP_305088 Tax_Id=9606 hypothetical protein XP_305088

**421.** EEDGC: 102336, >IPI:IPI00307216.1|REFSEQ_NP:NP_059125|TREMBL:Q9NVH5;Q9HAY6|ENSEMBL:ENSP00000258168 Tax_Id=9606 Beta, beta-carotene 15,15'-dioxygenase

**422.** DECEG: 102526, >IPI:IPI00337828.1|TREMBL:Q9BXY3 Tax_Id=9606 Thrombospondin

**423.** DEEGC: 102716, >IPI:IPI00307298.1|TREMBL:O75197 Tax_Id=9606 Lipoprotein receptor related protein 5

**424.** ECEDG: 102922, >IPI:IPI00336105.1|ENSEMBL:ENSP00000332419;ENSP00000332864 Tax_Id=9606

**425.** CEDGE: 103100, >IPI:IPI00220106.1|REFSEQ_NP:NP_066016|TREMBL:Q9HCD8;Q9BWY0;Q9HBC4|ENSEMBL:ENSP00000304604 Tax_Id=9606 Membrane-associated guanylate kinase MAGI3

**426.** EDGEC: 103120, >IPI:IPI00307359.1|REFSEQ_NP:NP_683707|TREMBL:Q9BRN2;Q8NB17;Q86YQ3|ENSEMBL:ENSP00000325220 Tax_Id=9606 oxysterol-binding protein-like protein 9 isoform f

**427.** EGEDC: 103162, >IPI:IPI00065186.1|REFSEQ_NP:NP_872328|TREMBL:Q96LR4|REFSEQ_XP:XP_087261|ENSEMBL:ENSP00000295569 Tax_Id=9606 Hypothetical protein FLJ25161

**428.** EGECD: 103220, >IPI:IPI00097257.2|SWISS-PROT:Q96M20|REFSEQ_NP:NP_543024|ENSEMBL:ENSP00000279046 Tax_Id=9606 Protein C20orf152

**429.** ECGED: 103328, >IPI:IPI00220045.1|SWISS-PROT:Q9NVU0-1|REFSEQ_NP:NP_060589|ENSEMBL:ENSP00000299853 Tax_Id=9606 Splice isoform 1 of Q9NVU0 DNA-directed RNA polymerases III 80 kDa polypeptide

**430.** ECGDE: 103714, >IPI:IPI00011245.1|SWISS-PROT:Q9HBJ7|REFSEQ_NP:NP_065954|ENSEMBL:ENSP00000254181 Tax_Id=9606 Ubiquitin carboxyl-terminal hydrolase 29

**431.** GEECD: 103878, >IPI:IPI00011224.2|SWISS-PROT:O43184-1|REFSEQ_NP:NP_003465|ENSEMBL:ENSP00000278082 Tax_Id=9606 Splice isoform 12L of O43184 ADAM 12 precursor

**432.** ECGDE: 103928, >IPI:IPI00007777.1|SWISS-PROT:Q9UL36-1|TREMBL:O95276|ENSEMBL:ENSP00000253159 Tax_Id=9606 Splice isoform B of Q9UL36 Zinc finger protein 236

**433.** ECGDE: 103930, >IPI:IPI00307551.1|SWISS-PROT:Q9UL36-2|REFSEQ_NP:NP_031371|ENSEMBL:ENSP00000322361 Tax_Id=9606 Splice isoform A of Q9UL36 Zinc finger protein 236

**434.** EDEGC: 104252, >IPI:IPI00013162.2|SWISS-PROT:P41217-1 Tax_Id=9606 Splice isoform 1 of P41217 OX-2 membrane glycoprotein precursor

**435.** EDEGC: 104260, >IPI:IPI00221361.1|SWISS-PROT:P41217-2|REFSEQ_NP:NP_005935|ENSEMBL:ENSP00000312766 Tax_Id=9606 Splice isoform 2 of P41217 OX-2 membrane glycoprotein precursor

**436.** CEDGE: 104728, >IPI:IPI00001152.1|REFSEQ_NP:NP_112168|TREMBL:Q9BYT3|ENSEMBL:ENSP00000252710;ENSP00000320754;ENSP00000320933 Tax_Id=9606 Serine/threonine kinase 33

**437.** EEGDC: 105006, >IPI:IPI00012165.3|SWISS-PROT:Q9HC84|TREMBL:Q93043;Q9NYE4|REFSEQ_XP:XP_039854;XP_039877|ENSEMBL:ENSP00000227189 Tax_Id=9606 Mucin 5B precursor

**438.** EECDG: 105502, >IPI:IPI00261059.1|REFSEQ_XP:XP_303959 Tax_Id=9606 hypothetical protein XP_303959

**439.** EEGDC: 105532, IPI:IPI00021119.1|REFSEQ_NP:NP_003645|TREMBL:O43916|ENSEMBL:ENSP00000309270 Tax_Id=9606 Chondroitin-6-sulfotransferase (Hypothetical protein)
